# Efficacy of Sphincter Control Training (SCT) in the treatment of premature ejaculation, a new cognitive behavioral approach: A parallel-group randomized, controlled trial

**DOI:** 10.1371/journal.pone.0212274

**Published:** 2019-02-26

**Authors:** Jesús E. Rodríguez, Juan C. Marzo, José A. Piqueras

**Affiliations:** 1 Murcian Institute of Sexology, Murcia, Spain; 2 Department of Health Psychology, University Miguel Hernández of Elche, Elche, Spain; Eberhard Karls Universitat Tubingen, GERMANY

## Abstract

**Introduction:**

Current evidence suggests that Cognitive Behavioral therapy (CBT) has a limited role in the contemporary management of premature ejaculation (PE).

**Aim:**

The aim of this study was to determine the efficacy of a new CBT for the PE called Sphincter Control Training (SCT) in combination with a masturbation aid device.

**Methods:**

The present study included 35 patients’ that met diagnostic criteria for PE including intravaginal ejaculatory latency time (IELT) of ≤2 minutes and had a Premature Ejaculation Diagnostic Tool (PEDT) score ≥11. Participants completed all phases of a randomized controlled clinical study with a parallel group design, which was approved by the Ethical Committee of the Hospital Morales Meseguer of Murcia (Spain).The two treatment groups completed SCT over 7 weeks. The SCT consists of four different exercises and an educational session. Its objective is to provide patients with greater knowledge, awareness, and control of the external urethral sphincter. The only difference between groups was the use of a masturbation aid device called Flip Zero (TFZ-001) from the Japanese company Tenga Co., Ltd.

**Outcomes:**

The main measure was the "fold increase" (FI) of the IELT, which was calculated using the geometric mean pre-treatment and post-treatment. In addition, Premature Ejaculation Profile PE was used as a secondary measure.

**Results:**

The geometric mean of the measurements corresponding to the 7 weeks of treatment was calculated, and both groups were compared by means of an ANCOVA test, finding a statistically significant difference (F: 10.51, 1; p = .003) in the increase experienced by subjects in the group with the device (GWD) mean = 166.63, SD = 106.54) compared with that experienced by subjects in the group without device (GWtD) (mean = 86.99, SD = 59.98).Using Student's t-test, the Fold increase (FI) corresponding to both groups were compared. The results showed statistically significant differences (p = .008) between the measurements corresponding to the GWtD (1.38 (0.50)) and those relative to the GWD (2.69 (1.81))

**Clinical implications:**

The FI in the GWD at the end of the trial allow us to consider this new CBT as a potential and viable PE treatment alternative. No side effects were observed in either treatment group and it required little therapeutic input and no partner involvement.

**Strengths & limitations:**

The main limitation of this study is the lack of a 3- to 6-month follow-up of the treatment and placebo control.

**Conclusions:**

This SCT exercise program combined with the use of a masturbation device shows promise because has numerous advantages in relation to current recommended treatments in patients with PE.

## Introduction

Premature ejaculation (PE) is the most common sexual dysfunction in men. Although new criteria and classifications in recent years have aimed to obtain a better definition of this disease, the true prevalence of PE remains unclear [1].

Regarding its treatment, the evidence to date confirms the efficacy and safety of dapoxetine (*level of evidence (LOE) 1a*), off-label selective serotonin reuptake inhibitors (SSRIs) (*LOE 1a*), and topical anesthetics (*LOE 1b*), which are considered first-line treatments according to the International Society for Sexual Medicine (ISSM) [2].

In relation to behavioral treatments (BTs), the evidence is very limited (*LOE 2b*), although results improve when drugs are combined with BT, in contrast to the exclusive use of drugs [3, 4].

Most studies investigating the efficacy of different BTs do not meet the standards of evidence-based medicine because they are non-randomized studies without a control group, use small samples without adequate follow-up, and/or use a variety of basic definitions of PE [5].

Despite this limitation, BTs may be a promising alternative in the management of PE. Their advantages include the absence of side effects, addressing aspects of PE not addressed by medication alone, and the potential to be able to maintain their results as learning outcomes, avoiding relapses by stopping the medication [5].

New BTs appear as a result of the technological development and popularity achieved by the sex toy industry worldwide in recent years. These treatment programs combine classic behavioral techniques such as the stop-start technique of Semans (1956) with the use of masturbation devices.

In the year 2000, Wise [6] mentioned the use of a vibratory desensitizing masturbation aid for the treatment of PE in a series of cases, where the mean increase in the intravaginal ejaculatory latency time (IELT) for each patient was 15.2 minutes after six weeks of use. The device, designed by Zamar one year earlier, particularly for desensitization, was based on the theory of penile hypersensitivity as the cause of PE. Its designer considered that an increase in the intensity and frequency of stimulation of a hypersensitive organ resulted in habituation [7].

In 2012, at the World Meeting on Sexual Medicine in Chicago, Zamar presented a clinical trial with 56 participants in which he repeated the methodology of Wise et al. [6] but with a new version of his vibratory masturbation aid, aiming to combine it with the stop-start technique of Semans [8] as an enhancing device. This trial has produced good results after a 6-week intervention, with 11-fold increases in intravaginal ejaculation latency time in 61% of patients. Treatment benefits were stable over at least 3 months after completed treatment.

Two years later, Jern [9] replicated the clinical trial by Zamar with a sample of 13 subjects. As a novelty, a 3- to 6-month follow-up was performed after treatment. The author indicated the impossibility of finding similar significant differences in magnitude as those found by Zamar between the control group and the IELT waiting list group; although he recognized that there were detectable improvements that remained after 3 and 6 months.

Rodríguez and Lopez [10] presented a series of cases replicating the study of Wise et al. [6] in which a new type of masturbation device was included as a novelty, designed to reproduce vaginal stimulation without vibration. The results indicated an average increase in IELT from 1.4 minutes to 10.43 minutes. In this case, the researchers explained their results based on a process of habituation of the receptors of the glans penis by repeated use and by the intense stimulation of nerve receptors caused by the device, in line with the postulates of Wise et al. [6]. However, of the 15 subjects studied, valid data could only be obtained from three participants in the post-treatment phase, which is a great limitation to their conclusions.

One year later, Rodríguez et al. published another series of cases, using a new device with slight modifications to that of their previous study. Of the 20 cases initially included in the study, the post-treatment measures of 18 participants were valid. The methodology was improved, and a clinical improvement criterion was established based on pre- and post-treatment outcomes of the Premature Ejaculation Profile (PEP) instead of the IELT measure. A significant percentage of men met the established criteria of clinical benefits: 83% in control over ejaculation, 72% in stress and interpersonal difficulties associated with ejaculation, and 33% in satisfaction with their coital relationships [11]. In this case, the authors explained the improvements found by having used the device for 6 weeks combined with the stop-start strategy established in the guidelines, thus abandoning the habituation hypothesis. They considered that ejaculation training using stimulation of the receptors of the glans similar to what would occur during intercourse favors the transfer of what was learned during masturbation to intercourse.

A recent review of this new line of treatment using masturbators in combination with behavioral techniques considers this therapeutic approach as a promising alternative to pharmacological treatments, which could not only obtain a better IELT but also give control to men with PE, with no side effects, and stable results over time [7].

At the same time, and as for other BTs, they note the need to develop randomized studies with control groups and larger sample sizes. It has also been stressed that it would be desirable to determine whether the clinical benefit obtained in PE with these treatments is attributable to the use of these devices *per se*, the behavioral techniques used, or a combination of both.

Consequently, the aim of the present randomized, controlled clinical trial with two parallel groups is to determine if a new protocol of cognitive-behavioral treatment for PE called SCT benefits from the use of a masturbation device for carrying out the individual exercises. This device is an updated version of the one used by Rodríguez and Lopez [11] with ergonomic improvements.

It was hypothesized that the participants in the SCT programme plus device group would achieve significantly more improvement in outcomes measures than the only SCT programme group.

## Methods

A CONSORT-revised 2010 compliant [12] randomized controlled trial (RCT) parallel group design was used to compare the SCT group and the SCT plus device group. The trial protocol data in S3 File was approved by the Ethical Committee of the Hospital Morales Meseguer of Murcia (Spain) (EST: 34/17) and is registered with the ClinicalTrials.gov (Identifier: CT03304808).

First registration attempt in ClinicalTrials.gov ID: NCT03304808 was in March 2017 but it was incomplete because we need the approve of the Ethical Committee of the Hospital Morales Meseguer of Murcia (Spain). The approve of this Ethic committee EST:34/17 was in 27 September 2017 and we began the recruitment patient in first week October 2017.

The authors confirm that all ongoing and related trials for this intervention are registered.

### Participant recruitment

Prior to the commencement of the trial, power calculations were conducted to determine the minimum sample size. As there was no research outlining the efficacy of SCT, power calculations were informed by published RCTs trial design on PE for psychotherapeutic intervention [13].

We therefore aimed to recruit a minimum sample of 24 participants to allow to detect population treatment differences with a statistical power of 0.80 and an alpha level set at P = 0.05. Participants were recruited through a health marketing campaign developed by a specialized company called Flint, using an advertisement strategy in AdWords and a landing page with the domain yocontrolo.org, which took place in the first week October 2017.

A total of 35 subjects from all over Spain, aged between 22 and 53 years of age (mean = 33.7 years, standard deviation (SD) = 8.9) were recruited Table 1

**Table 1:** Baseline demographic data (*mean ± s*.*d*) in premature ejaculation patients who completed all phases of a randomized controlled clinical study.

	GWtD	GWD	*p-value*
**Number of patients**	17	18	
**Age (year)**	33(9.59)	33.11(8.91)	.97
**Duration of relation (month)**	74.18(88.41)	94.17(81.38)	.69
**IELT mean**	65.35(41.02)	70.17(34.64)	.71

GWtD: exercise program; GWD: exercise program + masturbation device. SD: standard deviation. p-value Student's t-test for independent samples

### The inclusion criteria

All participants met the following inclusion criteria: Aged over 18 years, being in a heterosexual relationship for at least the last 6 months, having a score of >11 on the Premature Ejaculation Diagnostic Tool (PEDT), and having a self-reported IELT ≤2 minutes.

### The exclusion criteria

Subjects were excluded if they reported: A history of alcohol abuse or dependence, having received medication or psychological treatment for PE in the last 6 months, having diabetes, or the regular use of recreational drugs (except tobacco and caffeine).

### Procedure

All subjects interested in participating who responded to the advertising campaign were contacted by email or telephone, and the questionnaires and selection records were sent by email. The subjects were asked for a two-week record of their IELT, and once received, they were administered the PEP questionnaire. Those who met the selection criteria were randomly assigned to the groups by recruitment order on a 1:1 basis and were asked to sign an informed consent before initiating treatment.

The subjects were free to leave the study at any time and did not receive financial compensation for participating, although the subjects of the experimental group that used a masturbation device were allowed to keep the device once the study concluded.

The two treatment groups completed Sphincter Control Training (SCT) Table 2 over 7 weeks.

**Table 2:** Sphincter control training programme.

Sphincter Control Training Method		
Timeline	Activity	Procedure
Week 1	"Discovering the pelvic floor"	Masturbation four times a week, paying attention to the pelvic muscles and external urethral and anal sphincters
Week 2, 3, 4	"External sphincter feedback with stop-start"	Masturbation four times a week, with four active stops per exercise for a maximum of 45 seconds, relaxing the external urethral and anal sphincters at each stop
Week 5, 6	"External sphincter feedback without cessation of stimulation"	Masturbation four times a week without cessation of stimulation, with four moments of relaxation by exercising the external urethral and anal sphincters before ejaculating
Week 7	"Feedback of the external sphincter with coital movements"	Masturbation four times a week without cessation of stimulation, with four moments of relaxation by exerting the external urethral and anal sphincters before ejaculating with coital movements

This exercise program was developed individually with all patients, and the only difference between groups was the use of a masturbation device called Flip Zeroby one of the treatment groups.

The SCT consists of four different exercises and an educational session data in S3 File. Its objective is to provide patients with greater knowledge, awareness, and control of the external urethral sphincter and its role in the ejaculatory reflex. The aim of these activities is that men learn to interfere in the ejaculatory reflex through the relaxation of the external sphincter, thus preventing the formation of the prostatic pressure chamber [14].

The subjects of both groups received an email with an educational PowerPoint presentation on the role of the external sphincter, files with each of the four SCT exercises accompanied by links to explanatory videos, and records for activity and IELT. Questions about the exercises could be addressed via email.

Once the subjects finished each activity and returned the completed records, they were emailed all the necessary materials for the next program activity.

Once the program was completed and the last activity record was received, the PEP questionnaire was administered again.

### Measures

#### PEDT

For the diagnosis, the PEDT was used, which measures five items assessing the following: difficulty in delaying ejaculation, ejaculation before the patient wishes, ejaculation with little stimulation, frustration related to PE, and opinion of the couple about the ejaculation. The test-retest reliability of the PEDT is .82, and all items discriminate in a statistically significant way between patients with PE and without PE, with a cut-off score for the diagnosis [15].

#### Fold increase IELT

The main measure was the "fold increase" (FI) of the IELT, which was calculated using the geometric mean of post-treatment IELTs (period B) divided by the geometric mean of pre-treatment IELTs (period A): FI = (geometric mean of IELT_7wk)/(geometric mean of PRE_IELT) [16].

For 7 weeks, the IELTs were collected through weekly record sheets, specially designed for the study. These were delivered with the rest of the audiovisual materials and files for each activity. These same record sheets were used for 2 weeks before the start of treatment and served to establish an IELT baseline for each subject (period A).

Participants received instructions, together with the first record, of how they should calculate the time elapsed between the beginning of the penetration and the moment in which they ejaculated, using their mobile phone stopwatch.

#### PEP

In addition, one of the patient-reported outcomes (PROs) recommended for PE was used as a secondary measure [17]; the subjects completed a questionnaire prior to the start of treatment and another at the end of the treatment. The PEP by Patrick et al. [18] is a self-reported instrument used to evaluate the domains of PE and its treatment. It consists of four measures (perceived control over ejaculation, personal stress related to ejaculation, satisfaction with sexual relations, and interpersonal difficulty related to ejaculation) that yield a profile and an overall score where the worst scores express the worst performance. The intraclass correlation coefficient (reliability) ranges from .66 to .83, and its validity differs with dapoxetine and IELTs. This instrument has been validated in the American and European populations of Germany, France, the United Kingdom, Italy, and Poland [19], although it has also been used with the Spanish population [20].

### Statistical analysis

First, to verify that there was no difference between the groups prior to treatment, the mean difference in the IELT of both groups was calculated using Student's t-test.

Analysis of covariance (ANCOVA) was performed to analyze the difference between the geometric means of group 1 (without masturbation device) and group 2 (with masturbation device) after the first 4 weeks, as well as after 7 weeks. The geometric mean was the dependent variable, treatment was considered as a factor, and the pre-treatment IELT as a covariate.

After that, to compare the progress of each group, intragroup comparisons were performed, using Student's t-test for related samples, between the previous values of IELT and its geometric mean at both 4 and 7 weeks.

As a complementary measure to verify the difference in the progress of both groups, Student’s t-test for independent samples was also used to compare the difference in the FI value between the two groups.

## Results

### Subjects

**Fig 1:**
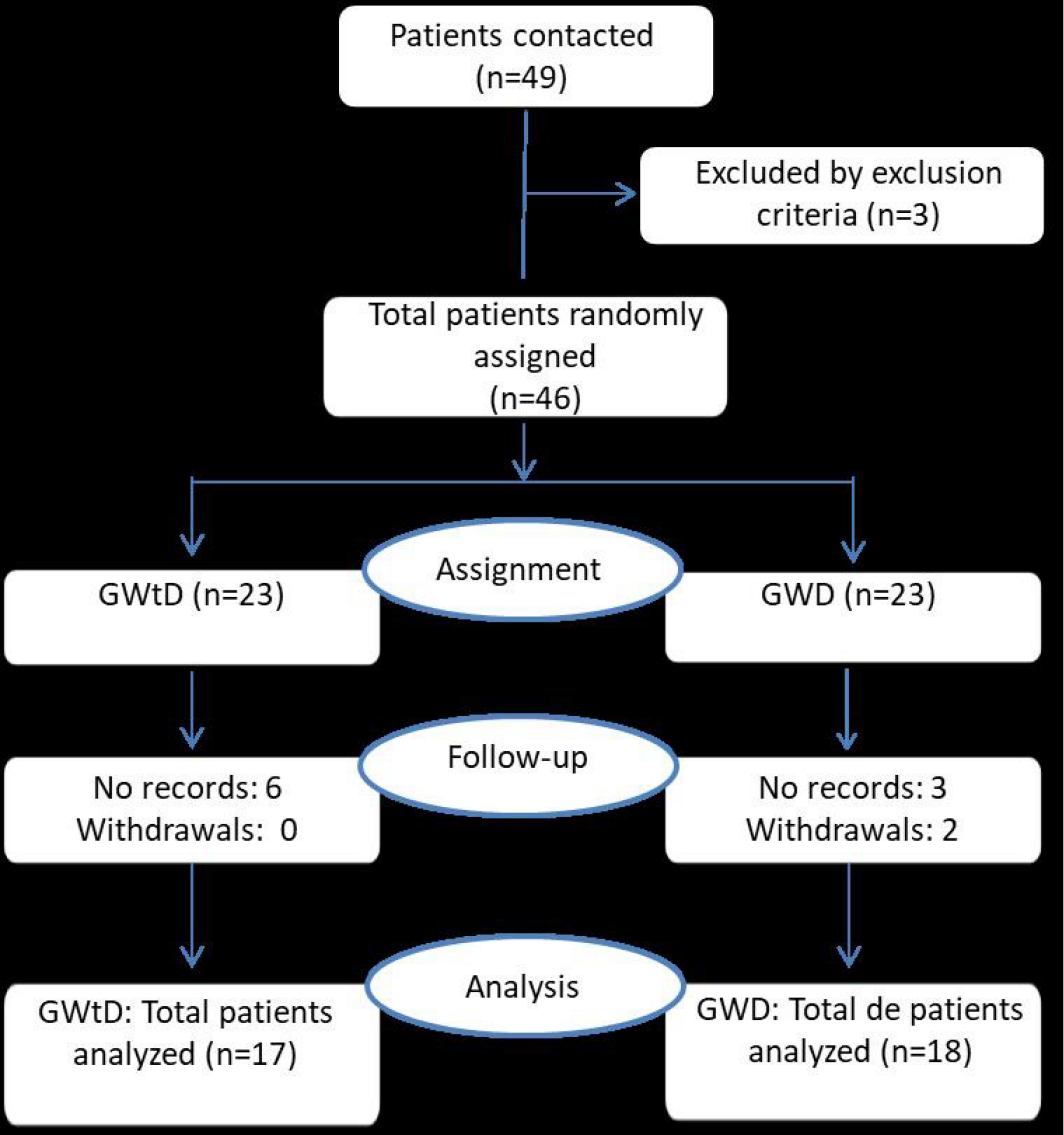
Flow diagram of participants. GWtD group: exercise program. GWD group: exercise program + masturbation device. Intravaginal ejaculatory latency time (IELT) and fold increase (FI).

Of a total of 49 subjects who were contacted, 46 were randomly assigned to treatment groups; 23 to the group without the device (GWtD) (exercise program), and 23 to the group with the device (GWD) (exercise program plus masturbation device). However, experimental mortality was high, as only 35 subjects completed all phases of the study and were included in the analysis, resulting in a mortality rate of 28.5%. The experimental mortality was similar in both groups. The non-delivery of records was the reason in 9 of the 11 subjects for leaving the trial. There were 17 subjects analyzed in the GWtD and 18 in the GWD (Fig 1).

The mean age for patients in the GWtD was 33.00 years (SD = 2.32), while the mean age for patients in the GWD was 33.11 years (SD = 2.10).

### IELT and fold increase

Table 3 shows the IELT values throughout the study. The pre-treatment mean of the GWtD was compared with that of the GWD using Student's t-test (t: -0.38, 33; p = .71), so it can be affirmed that there were no significant differences between the two groups before treatment.

**Table 3:** Intravaginal ejaculatory latency times (IELT) and fold increase (FI) during the study.

		GWtD Group	GWD Group	*p-value*
**IELT** **Geometric mean (SD)**	Pre-treatment (prtr)	65.35 (41.02)	70.17 (34.64)	.71
	4 weeks (4 wk)	75.10 (52.58)	140.45 (94.70)	.06†
	*p*-value (prtr/4 wk)	.105ⱡ	.002ⱡ	
	7 weeks (7 wk)	86.99 (59.98)	166.63 (106.54)	.003†
	*p*-value (4wk/7wk)	.001ⱡ	.001ⱡ	
**FI**	7 weeks (prtr/7 wk)	1.38 (0.50)	2.69 (1.81)	.008ⱡ

†Analysis of covariance

ⱡ Student's t-test for related samples

GWtD: exercise program; GWD: exercise program + masturbation device. SD: standard deviation

Next, the geometric mean corresponding to the first 4 weeks of treatment (IELT_4wk) for both groups was compared by means of an ANCOVA test, finding a statistically significant difference (F: 8.84, 1; p = .006) in the increase in IELT experienced by the subjects in the GWD compared to that experienced by subjects in the GWtD.

Subsequently, the geometric mean of the measurements corresponding to the 7 weeks of treatment was calculated, and both groups were compared by means of an ANCOVA test, finding a statistically significant difference (F: 10.51, 1; p = .003) in the increase experienced by subjects in the GWD (mean = 166.63, SD = 106.54) compared with that experienced by subjects in the GWtD (mean = 86.99, SD = 59.98).

The differences between pre-treatment values (PRE_IELT) and values after 4 weeks of treatment (IELT_4wk) were also analyzed for both groups using Student's t-test. The results showed that, in the case of the GWtD, there was not a statistically significant difference (p = .105) between either measurement. However, we found statistically significant differences (p = .002) between both measurements for the GWD.

Next, the differences between the values corresponding to the first 4 weeks of treatment (IELT_4wk) versus the values after the end of treatment (IELT_7wk) were analyzed for both groups using intragroup comparisons. The results showed that in both the GWtD (p = .001) and the GWD (p = .001), there were significant differences between the pretest and posttest.

Next, the variable named FI was created, which relates the measurements obtained during the weeks prior to treatment (PRE_IELT) with those obtained after 7 weeks of treatment (IELT _7wk) by means of the expression: FI = (geometric mean of IETL_7wk)/(geometric mean of PRE_IELT).

Using Student's t-test, the FIs corresponding to both groups were compared. The results showed statistically significant differences (p = .008) between the measurements corresponding to the GWtD and those relative to the GWD.

### Premature Ejaculation Profile (PEP)

**Fig 2:**
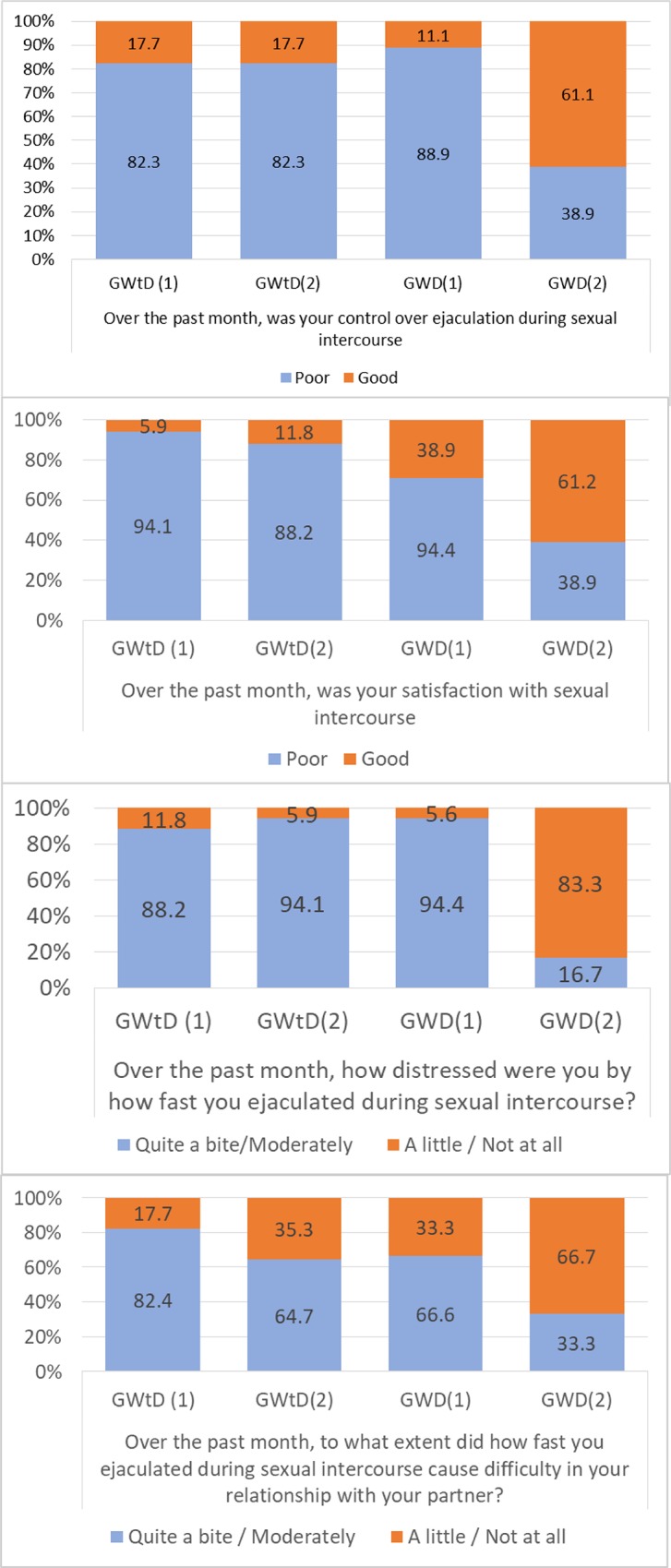
PEP frequencies. Response frequencies of the pre- and post-treatment Premature Ejaculation Profile (PEP).

Fig 2 shows the different response frequencies of the pre- and post-treatment PEP items for both groups. To represent the data with greater clarity, the percentages of subjects who answered "poor" or "regular", and "good" or "very good" in response to item 1 of control over ejaculation and item 2 of satisfaction during intercourse were grouped for the GWtD and the GWD. In the same way, the percentages of subjects who answered "moderately" or "quite", and "not at all" or "a little" in response to item 3 of perceived stress on ejaculatory speed and item 4 of affecting the couple relationship were grouped for the GWtD and the GWD by ejaculatory latency.In the GWD, the progress between pre- and post-treatment was very positive, something that did not happen in the GWtD.

Once the frequency tables were obtained, we calculated Pearson's chi square and found a relationship between variables only at post-treatment for each of the four PEP items at a significance level less than .05, and always in subjects in the GWD.

To calculate the strength of this relationship we used Cramer's V with values of .54 for item 1, .65 for item 2, .86 for item 3, and .48 for item 4.

## Discussion

This is the first randomized clinical trial using a parallel group design to measure the efficacy of a cognitive-behavioral treatment for PE that includes IELT measures and PROs [4].

The IELT geometric means showed, already from week four of the intervention, a superiority of SCT in combination with the use of the masturbation device compared with the GWtD.

This superiority was maintained until the end of the treatment at week 7, where, although significant improvements in IELT were observed for both groups over pre-treatment, there were still significant differences between the two groups.

During the study, no side effects were observed in either treatment group, which is a great advantage in relation to oral SSRI treatments because drug side effects are one of the main causes of their abandonment [21].

**Fig 3:**
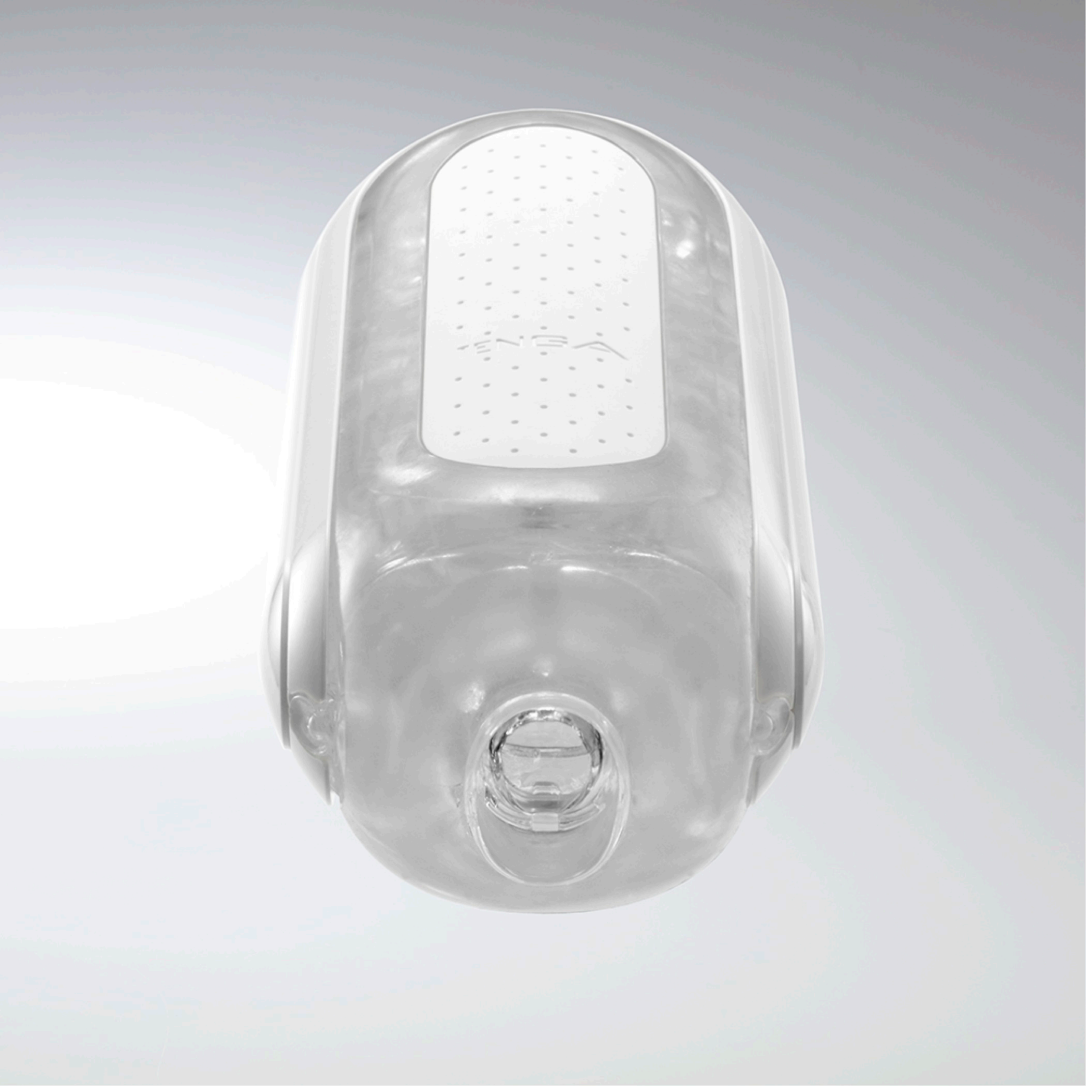
Masturbation device. TFZ-001 Flip Zero device (TENGA Co., Ltd, Tokyo, Japan). Reusable masturbation device of 70×80×180 mm.

The differences found between the two treatment groups can be attributed to the use of the masturbation device (Fig 3).

Patients with PE and IELT <2 minutes referring an adequate control of ejaculation during masturbation are commonly found in clinical practice. Being able to develop a self-control training program based on masturbation, although under similar conditions to coitus—given the type of stimulation produced by the masturbation device—would make it possible to more easily transfer what was learned to coital relationships by helping men develop greater control, as reflected in the response frequencies in item 1 of the PEP at the end of treatment in the GWD.

Although the subjects of the study had a stable partner, the SCT program was developed individually and online without the need for collaboration on the part of the couple, which would allow it to be used in patients with PE without a stable partner or for those who are reluctant to include their partners in the treatment or consult a health professional.

The main limitation of this study is the lack of a 3- to 6-month follow-up of the treatment groups and a placebo control. We cannot determine the need to continue with the practice of exercises to maintain the improvement found, both in IELT and perceived control. This question is important because the need for continuous use is another of the main reasons for the abandonment of current treatments with SSRIs [21].

Multicenter studies with larger sample sizes, appropriate follow-up and group control will be necessary to provide greater evidence of this new treatment strategy for PE.

## Conclusions

We can conclude that the new cognitive-behavioral strategy for the treatment of PE in which the SCT exercise program is combined with the use of a masturbation device achieve significantly more improvement in outcomes measures than the SCT alone. This combination shows promise although the efficacy cannot be assessed definitely.

## Supporting information

S1 File IELT registration sheet. This is the record of IELT using during the trial for the subjects in english.
(DOC)

S2 File IELT registration sheet. This is the record of IELT using during the trial for the subjects in Spanish.
(DOC)

S3 File Flip zero protocol. Study protocol Trial ISM-FLI-2017-01 in english.
(PDF)

S4 File Flip zero protocol. Study protocol Trial ISM-FLI-2017-01 in spanish.
(PDF)

S5 File Consort 2010. Checklist of information to include when reporting a randomised trial.
(DOC)
