# Supplementary material for: Efficacy of Sphincter Control Training (SCT) in the treatment of premature ejaculation, a new cognitive behavioral approach: A parallel-group randomized, controlled trial
Source: PLoS One. 2019 Feb 26;14(2):e0212274. doi: 10.1371/journal.pone.0212274 (PMC6391003; doi:10.1371/journal.pone.0212274)
Supplement: S1 File — This is the record of IELT using during the trial for the subjects in english. (DOC) [file pone.0212274.s001.doc]

TIME EJACULATION RECORD          WEEK:

NAME:


MONDAY
TUESDAY
WEDNESDAY        THURSDAY         FRIDAY

SATURD AY

SUNDAY


MASTURBATION


Indicate the time that is late in masturbating


INTERCOURSE
COITUS


Indicate the time that is late in ejaculating after penetrating


www.isemu.es
