# Supplementary material for: Efficacy of Sphincter Control Training (SCT) in the treatment of premature ejaculation, a new cognitive behavioral approach: A parallel-group randomized, controlled trial
Source: PLoS One. 2019 Feb 26;14(2):e0212274. doi: 10.1371/journal.pone.0212274 (PMC6391003; doi:10.1371/journal.pone.0212274)
Supplement: S2 File — This is the record of IELT using during the trial for the subjects in Spanish. (DOC) [file pone.0212274.s002.doc]

REGISTROS TIEMPO DE EYACULACION          SEMANA:

NOMBRE:


LUNES       MARTES       MIERCOLES    JUEVES            VIERNES        SABADO       DOMINGO


MASTURBACION


Indique el tiempo que tarda en eyacular cuando se masturba


PENETRACION
COITO


Indique el tiempo que tarda en eyacular despues de  penetrar


www.isemu.es
