# Supplementary material for: Efficacy of Sphincter Control Training (SCT) in the treatment of premature ejaculation, a new cognitive behavioral approach: A parallel-group randomized, controlled trial
Source: PLoS One. 2019 Feb 26;14(2):e0212274. doi: 10.1371/journal.pone.0212274 (PMC6391003; doi:10.1371/journal.pone.0212274)
Supplement: S3 File — Study protocol Trial ISM-FLI-2017-01 in english. (PDF) [file pone.0212274.s003.pdf]

1. IDENTIFICATION OF THE PROTOCOL

Research code:
ISM-FLI-2017-01

2. TITLE OF THE PROTOCOL

FLIP ZERO STUDY PROTOCOL
(Lifeflong Premature Eyaculation treatment)

3. IDENTIFICATION OF THE RESEARCHER.

RESEARCHER:

Jesús E. Rodríguez Martínez
Ph Student
Director of the Murcian Institute of Sexology. Murcia, Spain

Jose A. Picazo Aroca
Ph Student
Research area of the Murcian Institute of Sexology. Murcia, Spain

Juan C. Marzo Campos
Director of Department of Health Psychology; University Miguel Hernández of Elche, Spain

José A. Piqueras2 Rodríguez
Titular Professor of the Department of Health Psychology; University Miguel Hernández of Elche, Spain

4. SPONSOR.

Mr. Jesús Eugenio Rodríguez Martínez
Murcian Institute of Sexology
C/ Periodista Encarna Sanchez 22,
30007, Murcia

5. SUMMARY.

5.1. Sponsor identification

Instituto Sexológico Murciano (ISM)
C/Periodista Encarna Sanchez ,22, P1 30007 Murcia
Teléfono: 868 94 14 18

5.2. Title *of the Clinic essay.*

A randomized control trial to compare the eficacy and safety of an exercise programme that use a masturbation aid device as a first line treatment in patients with lifelong Premature Ejaculation (PE)

5.3. *Protocol code.*

Protocol code: ISM-FLI-2017-01

5.4. Principal researcher.

Mr. Jesús Eugenio Rodríguez Martínez
Director Insituto Sexologico Murciano
C/ Periodista Encarna Sanchez 22,
30007, Murcia

5.5. *Centers in those who are foreseen to realize the study*.

The essay is going to developed at the next sanitary centre:

1. **Instituto Sexológico Murciano**
C/ Periodista Encarna Sanchez Nº 22, P1 Nº
2. 30007 Murcia. Tel. 868 94 14 18 RES:
40000319

5.6. CEIC of reference and centres participants.

This essay has been submitted to the approval of the ethical .
committee of following reference:

- José Mª Morales Meseguer Hospital – Murcia

In this study the following centres will take part:

-Murcian Institute of Sexology-Murcia

5.7. Main Objective.

To compare the efficiency and safety of the use of a new program of exercises the one that is in use a device of help to the masturbation in the treatment of the primary premature ejaculation in Spanish population.

5.8. Desing.

This project of research will use two experimental groups, to one there managed the affairs the therapy "star-stop sign" (experimental 1) and other one the therapy "star-stop sign" combined with the external device (experimental 2). The subjects of both groups will receive a therapy of parallel form and with the same protocol of exercises (with the unique difference of which in the experimental one 2 the exercises of stop and take-off will be done by the external device). The assignment to each of the conditions will be random on order of entry to the study of those that were expiring with the criteria of selection.

5.9. Disease or disorder in study.

Lifelong Premature Ejaculation (F52.4).

5.10. *Information on the device object of study and the program of exercises:*

Device object of study : Flip Zero.

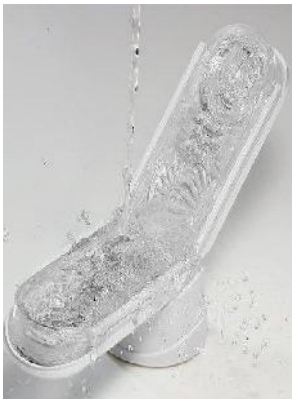

Flip Zero TFZ-001 , TENGA Co., Ltd.

Device of help to the masturbation hypoallergenic and with antibacterial reusable treatment.

Size: 70×80×180mm / (D)×(W)×(H)
Size in packaged: 77×92×213mm / (D)×(W)×(H)
Weigh: 460g
Other Information: reusable + 100 uses.
Instructions manual: annex 2

Exercises Program: Star-Stop 3.0.

The program of exercises to realize in house consists of 4 activities:

The first exercise called " Discovering the pelvic floor It will realize for one week, the second one named " Stop and Take-off " and the third party called had to be realized for 3 weeks," Stop and Take-off without cessation of stimulation " it was realized during 2 Weeks and last exercise of the program was naming " Stop and Take-off without cessation of the stimulation with coital movements " that It was developing for one week.

You register exercises on the annex 3

Lubricant used for the research: HOLE LOTION REAL.

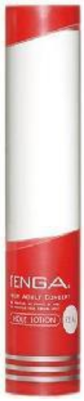

HOLE LOTION [REAL]: TLH-002 de TENGA Co., Ltd.

Package size: 44×44×177mm / (D)×(W)×(H)
Volumen 170ml.

Ingredients: wáter, glicerin, etanol, poliacrilato of sodium, hidroxicelulosa, fenoxietanol, EDTA-2Na, parabeno, polisorbato 80, cocoato of sorbitán, fragrance.

5.11. Population in study and total number of subjects.

The population in study it will be 18-year-old heterosexual men or more of age, of the whole national territory, which suffer lifelong premature ejaculation, which they have not received any previous treatment and which they have stable couple at least 6 months before beginning the essay.

There is foreseen a sample size of 50 patients. There are calculated 5 % of abandons or breach during the study.

5.12 Calendar and date foreseen of ending.

Duration period of incorporation: 2016 October-2017 June.
Date foreseen I initiate of the study: 2017 July.
Foreseen final date of the study: 2017 September.

5.13. Funding Source.

The financing will be chargeable to the area of research of the Murcian Institute of Sexology and to the Japanese multinational TENGA Co.

6. RELEVANT ASPECTS ON THE FINANCING OF THE STUDY.

The 70 % of the financing of the project will be chargeable to the area of research of the Murcian Institute of Sexology, whereas other one 30 % will be financed on the part of the Japanese company TENGA Co.

This 30 % includes the transfer of 30 devices Flip Zero TFZ-001 and of 30 boats of lubricant HOLE REAL LOTION: TLH-002 this way as the production and recording of a video with the different exercises of the program of treatment.

ECONOMIC MEMORY OF THE EXPENSES FORESEEN OF THE PROJECT OF RESEARCH:

Title: **FLIP ZERO PROTOCOL OF RESEARCH.**
Research code: **ISM-FLI-2017-01**

Principal researcher: **Jesús Eugenio Rodríguez Martínez**
Sponsor: **Murcian Institute of Sexology**

DETAILED DESCRIPTION OF THE EXPENSES FORESEEN WITH POST(CARGO) TO THE PROJECT

**STAFF COST:**	
1 Scholar area of research middle day	400,00 €/mes * 3
SUBTOTALS PAYMENTS:	1.200 ,00 €
**FACILITIES AND MATERIALS, LICENSES OF SOFTWARE AND TEST:**	
Licenses and kit of correction MCMI-III, PEP, PEDT and GRISS male.<br><br>1 Monthly license IBM spss stadistics software.<br>Sending agency national<br><br>MRW ecommerce transports 30 kit<br>Treatment.<br><br>Recording, edition and video production exercises Conrad Son media<br><br>Average	66,49 €<br>88,50 €<br>275,10 €<br><br>1.800,00 €
SUBTOTAL FACILITIES AND MATERIALS:	2.230,9 €
**CONSUMABLE ITEMS:**	
Customs taxes 30 KIT (HOLE LOTION [REAL]: TLH-002 y Flip Zero TFZ-001)<br>Declared value 30 KIT (HOLE LOTION + FLIP ZERO)	27,51 €<br>131,02 €
MATERIAL SUBTOTAL FUNGIBLE:	158,53 €

1

ECONOMIC MEMORY
Research code: ISM-FLI-2017-01

**ANOTHER COST :**	
Sanitary marketing campaing capture of subjects (Announcements and seekers)Flint Spain S.L.	1.875,50 €
Domain and hosting, research web, yocontrolo.org, Arsys S.L.U.	101,41€
Web template .Web Templates Ltd	67,00 €
Taxes of CEIC Morales Meseguer	669,63 €
SUBTOTAL ANOTHER COST:	2.713,54 €
**TOTAL PROJECT BUDGET:**	**6.302,97 €**

7. CENTRE WHERE ONE IS GOING TO DEVELOP THE ESSAY

The study is going to develop in the Instituto Sexológico Murciano (ISM) sanitary private centre.
Type of centre and Code: C.2.2 Consultation of other sanitary professionals.

Authorized services and code: Or 900.1 OTHER UNID.ASIST.PSICOLOGIA

Address: Street Journalist Personifies Sanchez. Floor number 1, n º 2, 22, Murcia.

Authorization of functioning: 09/10/2013

Inscription in the Record of the Warden ship of Health with the N º: 40000319

8. JUSTIFICATION AND RELEVANCY OF THE ESSAY

The premature ejaculation (EP) understood as a condition in which short (TLEI) gives itself a mistake of ejaculation control accompanied of times of intravaginal ejaculatory latency (of up to a minute), with a minimal stimulation and before the person wishes it (Serefoglu et to., 2014) is considered more frequent sexual masculine dysfunction, since it affects million men in the whole world (Russo et to., 2016). At present the treatments for EP consists of a combination of medicaments and sexological therapies or psychological, especially behavioural skills for the man and his couple.
The psychological theories try to explain the etiologic of the EP from a point of Sight that includes the effect of the early experience and the sexual conditioning, the anxiety, the sexual skill, the frequency of the sexual activity, and psychodynamics explanations of the development (p.e, Brody and Weiss, 2015).
Nevertheless, they are the biological theories those that possess more scientific evidence (p.e, Waldinger, 2002, Crown et to., 2008). Of between these, the classic model explains the human process of ejaculation in that the issue, with the training of a " chamber of pressure " (created in the prostate urethra), is followed by the expulsion (rhythmic contractions of the muscle spongy bulb) of the seminal fluid (Marberger, 1974).
Though the new ones and, often better sold pharmacological therapies are eclipsing the psychological and behavioural traditional methods in the treatment of the EP, today there is no evidence of which in the base of the EP only there is a physiological reason (Althof et to., 2014). In addition, there has been verified that the psychological strategies, based on behavioural skill, for the treatment of the EP have had certain success in the relief of this dysfunction (Althof et to, 2006).
Only a few studies have compared the psychotherapeutic skill for the EP, already be opposite to a condition of control (that stays in waiting-list) or against another psychological treatment. The mistake of specific protocols of treatment and of funds of research to carry out studies of checking well designed of these protocols has diminished the attraction of these approaches in relation to the evolution of the pharmacological strategies.

In spite of the fact that several cognitive behavioural strategies approach the fulfilment of the criteria of empirical support. Between them, the method of "start-stop sign", developed by Semans (1956) and adapted by other authors like Glina et to (2007), suppresses the urgency of ejaculating stopping or interrupting the sexual stimulation before coming to the point of imminent ejaculation by means of a squeeze of the glans penis. In addition, it is known that the use of external devices can turn out to be effective to help to control the ejaculation (Rodríguez and Lopez, 2016).

This project tries to demonstrate the efficiency on the EP of the combined treatment "star-stop sign" adapted of Semans (1956) in the frame of a program of exercises carried out with an external device similar to the employee in Rodríguez and Lopez (2016). The study tries to contribute scientific and methodological evidence to a cognitive - behavioural treatment offering an alternative to the pharmacological current treatments for the EP.

9. JUSTIFICATION AND DESING

This project of research will use two experimental groups, to one there will manage the affairs the therapy "star-stop sign" (experimental 1) and another one the therapy "star-stop sign" combined with the external device (experimental 2). Both groups will receive therapy of parallel form and with the same protocol of exercises (with the unique difference of which in the experimental one 2 the exercises of stop and take-off will be done by the external device. The assignment to each of the conditions will be random on order of entry to the study of those that were expiring with the criteria of selection.

In both groups, and to allow that both groups of subjects could accede to the treatment for the EP, the control is intra-fastened. It is going to pick up, during a similar period to that of treatment, measures of the principal variables that will be used to establish the line bases of every group.

10. MAIN OBJETIVE.

The aim of the project is to demonstrate the efficiency on the EP of the combined treatment "star-stop sign" adapted of Semans (1956) in the frame of a program of exercises carried out with an external device similar to the employee in Rodríguez and Lopez (2016).

The efficiency on the EP will see (TLEI) reflected in an increase in the times of latency of intravaginal ejaculation and in punctuations of subjective perception, of the therapy on the line base. The major efficiency of the treatment combined ("star-stop sign" together with external device) will meet reflected in the major increase of these TLEI and in punctuations of subjective perception of the therapy combined on the therapy "star-stop sign". The level of scientific evidence will mark the competitiveness of this psychological cognitive-behavioural skill.

11. MAIN OUTCOME MEASURE

The principal variable of measure is the time of latency of intravaginal ejaculation (TLEI). This one is a variable that calculates the own experimental subject and is the time, in seconds, since it begins the penetration until he ejaculates. It is a question of the variable most studied in researches of this type (Waldinger, Zwinderman, Olivier, and Schweitzer, 2008).
Other variables that are born in mind are of nature more psychological and of character auto informed by the own patients: On the one hand, the perception that the subjects have on his own ejaculation: the personal stress that provokes them, the satisfaction with the sexual relations and the interpersonal difficulty related to the ejaculation and, for other one, variables related to the sexual satisfaction as the perception of impotence, perception of premature ejaculation, avoidance of the sexual relations, mistake in sensuality and level of satisfaction / dissatisfaction with it sexual relations.

12. POPULATION OF STUDY AND TOTAL NUMBER OF PATIENTS

The subjects that will be born in mind will be all the interested parties in informing that their answer to the campaign of national advertising that will be started. These will be contacted by e-mail or telephonic route, the questionnaires and records of selection will be sent by e-mail (the criterion of incorporation in the study is defined in the following paragraph). Also there will be able to take part in the study all those patients who come to the ISM requesting evaluation and treatment for the EP and that fulfill the criteria of incorporation. The subjects will be free to leave the study at any time and will not receive economic compensation for taking part, though they will receive a gift in conclusion of the study, a high rate of abandons is foreseen.

On the basis of studies randomized to value the efficiency of treatments for the premature ejaculation that group of control existed, we think that it will be necessary 24 subjects for group to establish differences considering a statistical power of 0.80 and one alpha of p=0.05

13. CRITERIA OF INCORPORATION AND PATIENTS' EXCLUSION

The criteria of incorporation to be selected will be: To have more than 18 years, to be in a heterosexual relation at least during the last 6 months, a major punctuation has that 11 in the PEDT (Premature Ejaculation Diagnostic Tool) and a TLEI (Time of latency intravaginal ejaculatory) auto informed way 120 seconds.

The criteria of exclusion will include: Record of abuse or dependence of alcohol, to have received mediation or psychological treatment for the EP in the last 6 months, to suffer diabetes or the habitual use of drugs (exempting tobacco and caffeine)

14. STATISTICAL ANALYSIS.

Since principal measure will be in use " fold it was uncreating " of the TLEI that will be calculated using the geometric averages of the TLEI post - treatment (period B) divided by the average of the TLEI on having begun the treatment that they were using as line bases (period A).

There will be in use the test of Mahalanobis's distance for Outliers's checking existence (in that the punctuation Z> 3) and Kolmogorov-Smirnov to verify the goodness of fit to the normality of the information and the transformed one in those cases in which the supposition of normality is not fulfilled

Greenhouse-Geisser's method will be in use for correcting the degrees of freedom when the supposition is not fulfilled of spherical. There will be realized the contrast MANOVA for the demographic variables, presumably we will find significant differences in variables as age.

For the contrasts there will be in use ANCOVAS of independent measures, with the variables that have worked out significant in the contrast MANOVA as co varied, for verifying the differences between both experimental groups and the equality among lines base in each of the variables of study. And, of similar form, ANCOVAS of measures repeated to verify the existence of the benefits of every therapy on the line bases.

All the values of PE will must be bilateral and the level of meaning used 0.05. There will be in use the statistical package IBM SPSS Statistic version 22.

15. ETHICAL CONSIDERATIONS.

This study will have to develop in agreement with the protocol and with the procedure of Good Clinical Practice (GCP), as it is described in: Tripartite Harmonized Procedure of the ICH for Good clinical Practice 1996.

The researcher agrees, with the company of this protocol, to continue the instructions and procedure described in the same one and therefore it will fulfil the beginning of Good Clinical Practice on which it is based bearing in mind the Declaration of Helsinki http: // www.unav.es/cdb/ammhelsinki2.html

All the patients will pass for a therapeutic condition, in case of the therapy "star-stop sign" the efficiency is verified by previous studies (Glina et to., 2007) and in case of the combined therapy, the risk for the health is minimal and the probability of his efficiency is like a minimum like the therapy "star-stop sign" since, in this case, the first one it includes the second one.

In addition, to the completion of the study a gift will be granted for those who conclude it valued between 17 and 92 € with a view to “bounce of 170 ml of lubricant " and " device of help to the masturbation ".

The quiet information will be subject to the protection law of information: organic Law 15/1999 of December 13 of Protection of Information of Personal Character (LOPD). And the informed assent is gathered from conformity by the Royal decree 223/2004 from February 6

.

16. TREATMENT DURATION.

The duration of the treatment will be 7 weeks during which a series of exercises will be practised previously explained.

17. EVALUATION AND SAFETY OF THE ESSAY.

The material used in the research will be innocuous and his responsible use does not produce adverse reactions of interest.

One will proceed to the creation of a file of information of personal character with ownership of Instituto Sexológico Murciano, with the purpose of the withdrawal of these and later study.

There will remain a copy of support of the information and of the procedures of recovery of the same ones in a different place from that one in which there are the equipments that treat them.

There will be an assignment to every subject of a numerical or alphanumeric code according to a scheme of random generated statistically.

The person in charge of the file or of the treatment guarantees that only he has access and treats the personal information.

The confidential information will be coded by an algorithm of coding and a key that makes them illegible if the above mentioned key is not known.

The not automated files will store in cupboards or file cabinets of documents, which in turn, in a place with access protected by means of doors with key.

The worn lubricant this one made with innocuous ingredients. There should not be problems in case of ingestion. Nevertheless, in case of irritation they will stop using it and will be excluded from the research). In case of allergies the users will be informed about the ingredients before its utilization and was indicated all that they should do a small test of tolerance in the arm before using it in the genital zone.

18. CALENDAR AND DATE FORESEEN OF ENDING

The period of selection and patients' incorporation to take part in the study is fixed between October, 2016 and June, 2017.

The date foreseen of beginning of the study and beginning of the treatments is on July, 2017. The date foreseen to finish with the program of exercises in both groups is estimated at the end of September, 2017.

19. SURVEYS, INTERVIEWS OR INSTRUMENTS THAT ARE IN USE IN THE ESSAY

TLEI's measure will be gathered across an own register for week of treatment (ANNEX 1)

For the withdrawal of information relative to the perception on the ejaculation, there will be in use the Premature Ejaculation Profile (PEP) (Patrick et to., 2009), an auto informed instrument that is clear of four measures (Control perceived on the ejaculation, personal stress related to the ejaculation, satisfaction with the sexual relations and interpersonal difficulty related to the ejaculation) that they throw a profile and a general punctuation (Patrick et to., 2009) where the worst punctuations express a worse performance. The coefficient of reliability between classes goes from 0.66 to 0.83 and its validity was confirmed by the dapoxetina and the Times of Latency of Ejaculation Intravaginal (TLEI). This instrument was validated on American and European population by Germans, Frenchman, English men, Italians and Poles (Giuliano et to., 2008) though it has been in use also in Spanish population (Bar-Or, Salottolo, Orlando, Winkler, and Group, 2012; Buvat, Tesfaye, Rothman, Rivas, and Giuliano, 2009).

For the diagnosis there will be used the Premature Ejaculation Diagnostic Tool (PEDT) that measures five articles that they evaluate: impede to retard the ejaculation, ejaculation before that the patient wishes it, ejaculation with few stimulation, frustration related on having ejaculated prematurely and opinion of the couple about the ejaculation. The reliability test - retest is of 0.82 and all the articles discriminate of statistically significant form between patients with PE and without PE.

Also one will administer the Inventory of Sexual Satisfaction of Golombok and Rust (GRISS, initials in English). It is a question of a brief questionnaire of 28 questions to evaluate the existence and gravity of the sexual problems of each one of the members of the heterosexual couples (Rust and Golombok, 1985, 1986). This questionnaire provides punctuations for men and women separately in 3 shared scales (avoidance, not sensuality and dissatisfaction) and other 6 specific ones of every sex (Impotence, premature ejaculation, anorgasmia, vaginism, seldomness and lack of communication) with a few acceptable values of reliability and validity (Rust and Golombok, 1985, 1986).

The instrument was validated in English population and already it is begun to use in some Spanish works of Sexologic's official master of the university of Almeria (García Gonzalez, 2013; Lopez Lorente, 2013).

The external device of help to the masturbation will be the Flip-Hole of the company TENGA, Co . Flip Zero TFZ-001 , TENGA Co., Ltd.

Device of help to the masturbation hypoallergenic and with antibacterial reusable treatment:

Size: 70×80×180mm / (D)×(W)×(H)
Packaged size: 77×92×213mm / (D)×(W)×(H)
Packaged weigh: 460g
Other dates: reusable + 100 use.

The lubricant used during the study names:HOLE LOTION [REAL]: TLH-002 de TENGA Co., Ltd.

Packaged size: 44×44×177mm / (D)×(W)×(H) Volume 170ml.
Ingredients: water, glicerin, ethanol, poliacrilato de sodio, hidroxicelulosa, fenoxietanol, EDTA-2Na, parabeno, polisorbato 80, cocoato de sorbitán, fragancia

20. REFERENCES

Althof, S. E., McMahon, C. G., Waldinger, M. D., Serefoglu, E. C., Shindel, A. W., Adaikan, P. G., … Torres, L. O. (2014). An Update of the International Society of Sexual Medicine's Guidelines for the Diagnosis and Treatment of Premature Ejaculation (PE). Sexual Medicine, 2(2), 60–90. https://doi.org/http://dx.doi.org/10.1002/sm2.28

Althof, S., Rosen, R., Symonds, T., Mundayat, R., May, K., & Abraham, L. (2006). Development and validation of a new questionnaire to assess sexual satisfaction, control, and distress associated with premature ejaculation. The Journal of Sexual Medicine, 3(3), 465–475.

Bar-Or, D., Salottolo, K. M., Orlando, A., Winkler, J. V, & Group, T. O. D. T. S. (2012). A randomized double-blind, placebo-controlled multicenter study to evaluate the efficacy and safety of two doses of the tramadol orally disintegrating tablet for the treatment of premature ejaculation within less than 2 minutes. European Urology, 61(4), 736–743.

Brody, S., & Weiss, P. (2015). Erectile Dysfunction and Premature Ejaculation: Interrelationships and Psychosexual Factors. The Journal of Sexual Medicine, 12(2), 398–404. https://doi.org/http://dx.doi.org/10.1111/jsm.12738

Buvat, J., Tesfaye, F., Rothman, M., Rivas, D. A., & Giuliano, F. (2009). Dapoxetine for the Treatment of Premature Ejaculation: Results from a Randomized, Double-Blind, Placebo-Controlled Phase 3 Trial in 22 Countries. European Urology, 55(4), 957–968. https://doi.org/10.1016/j.eururo.2009.01.025

Corona, G., Jannini, E. A., Mannucci, E., Fisher, A. D., Lotti, F., Petrone, L., … Forti, G. (2008). Different testosterone levels are associated with ejaculatory dysfunction. The Journal of Sexual Medicine, 5(8), 1991–1998.

García González, S. (2013). Deseo, autoestimulación, satisfacción y fantasías sexuales en personas con necesidades especiales. Universidad de Almería. Retrieved from http://repositorio.ual.es/bitstream/handle/10835/2542/Trabajo.pdf?sequence=6

Giuliano, F., Patrick, D. L., Porst, H., La Pera, G., Kokoszka, A., Merchant, S., … Polverejan, E. (2008). Premature Ejaculation: Results from a Five-Country European Observational Study. European Urology, 53(5), 1048–1057. https://doi.org/10.1016/j.eururo.2007.10.015

Glina, S., Abdo, C. H., Waldinger, M. D., Althof, S. E., Mc Mahon, C., Salonia, A., & Donatucci, C. (2007). Premature ejaculation: A new approach by James H. Semans. Journal of Sexual Medicine, 4(4), 831.

López Lorente, A. M. (2013). Las relaciones de apego y su influencia en la satisfacción sexual de enfermos/as alcohólicos/as. Universidad de Almería. Retrieved from http://repositorio.ual.es/bitstream/handle/10835/2365/Trabajo.pdf?sequence=1&isAllowed=y

Marberger, H. (1974). The mechanisms of ejaculation. In Physiology and genetics of reproduction (pp. 99–110). Springer.

Patrick, D. L., Giuliano, F., Ho, K. F., Gagnon, D. D., McNulty, P., & Rothman, M. (2009). The Premature Ejaculation Profile: validation of self-reported outcome measures for research and practice. BJU International, 103(3), 358–364. https://doi.org/10.1111/j.1464-410X.2008.08041.x

Rodríguez, J. E., & López, A. (2016). Male masturbation device for the treatment of premature ejaculation. Asian Pacific Journal of Reproduction, 5(1), 80–83.

Russo, A., Capogrosso, P., Ventimiglia, E., La Croce, G., Boeri, L., Montorsi, F., & Salonia, A. (2016). Efficacy and safety of dapoxetine in treatment of premature ejaculation: an evidence-based review. International Journal of Clinical Practice, 70(9), 723–733.

Rust, J., & Golombok, S. (1985). The Golombok-Rust Inventory of Sexual Satisfaction (GRISS). British Journal of Clinical Psychology, 24(1), 63–64. https://doi.org/10.1111/j.2044-8260.1985.tb01314.x

Rust, J., & Golombok, S. (1986). The GRISS: a psychometric instrument for the assessment of sexual dysfunction. Archives of Sexual Behavior, 15(2), 157–165.

Semans, J. H. (1956). Premature ejaculation: A new approach. Southern Medical Journal, 49(4), 353–358.

Serefoglu, E. C., McMahon, C. G., Waldinger, M. D., Althof, S. E., Shindel, A., Adaikan, G., … Torres, L. O. (2014). An Evidence-Based Unified Definition of Lifelong and Acquired Premature Ejaculation: Report of the Second International Society for Sexual Medicine Ad Hoc Committee for the Definition of Premature Ejaculation. Sexual Medicine, 2(2), 41–59. https://doi.org/http://dx.doi.org/10.1002/sm2.27

Waldinger, M. D. (2002). The neurobiological approach to premature ejaculation. The Journal of Urology, 168(6), 2359–2367.

Waldinger, M. D., Zwinderman, A. H., Olivier, B., & Schweitzer, D. H. (2008). Geometric mean IELT and premature ejaculation: appropriate statistics to avoid overestimation of treatment efficacy. The Journal of Sexual Medicine, 5(2), 492–499

) SIGNATURE SPONSOR

INSTITUTO SEXOLOGICO MURCIANO
N.R.S 20300055
Tel [illegible] 94 14 1[illegible]
[illegible]

) PRINCIPAL INVESTIGATOR

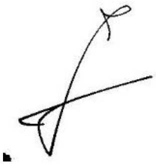

Annex 1

.

	LUNES	MARTES	MIERCOLES	JUEVES	VIERNES	SABADO	DOMINGO

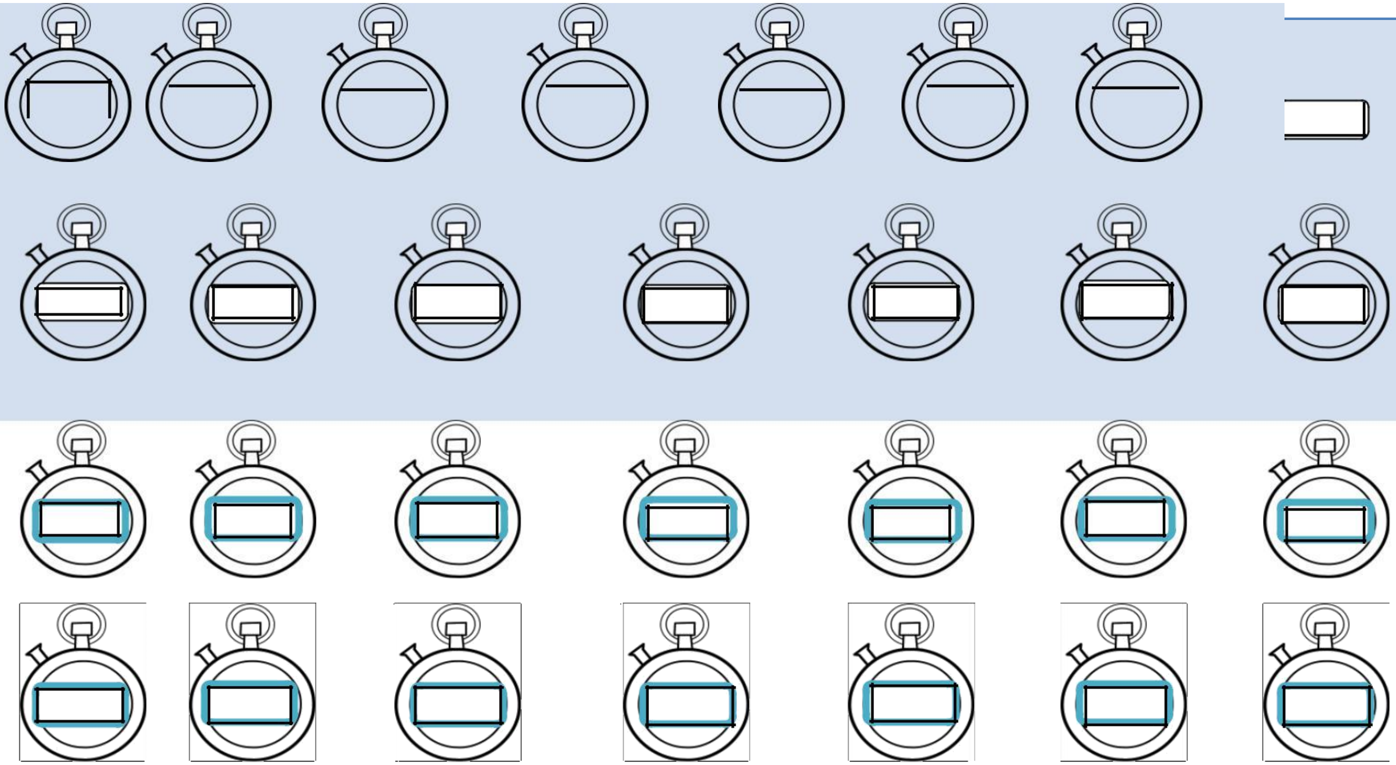

MASTURBACION

Indique el **tiempo que tarda en eyacular** cuando se **masturba**

PENETRACION COITO

Indique el **tiempo que tarda en eyacular** despues de **penetrar**

Annex 2

FLIP ZERO 0

USER MANUAL

English

Zeroing in on the Possibilities of Pleasure

TENGA has taken its unique FLIP Series tonew heights of pleasure.

A seamless insertion point provides the perfect seal for no lubricant leakage and a strong vacuum made possible by a one-way valve.

The FLIP 0 (ZERO) features our most intricate interna details yet for great sensation, and our pioneering FLIP-open function keeps the item hygienic for multiple uses.

Parts

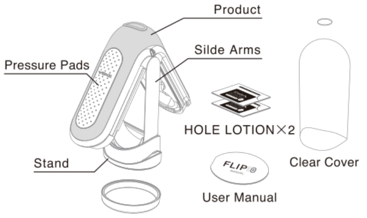

How to Open (1)

Remove Slide Arm

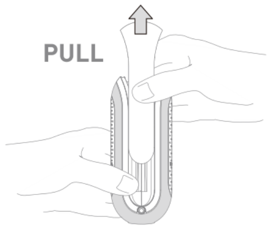

How to Open (2)

Squeeze Rails until lock clicks out of place.

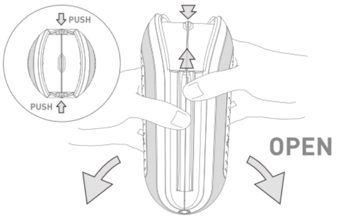

Before Use

Lubricate the interior of the FLIP ZERO.
Close and lock into place with Slide Arms, and lubricate insertion point.

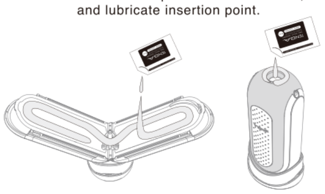

※We recommed TENGA Brand HOLE LOTION for further uses (sold separately.)

Using Pressure Pads

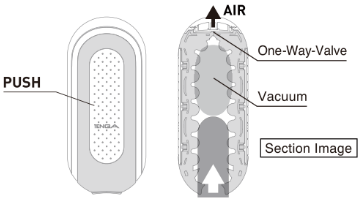

After insertion, you can squeeze the Pressure Pads at any point to provide focused stimulation and create an internal vacuum.

Internal Structure

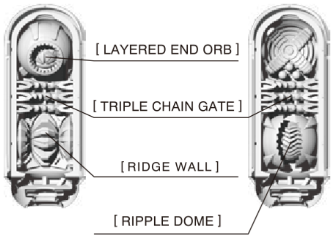

How to Dry

Remove Slide Arms and open Product.
Wash thoroughly with warm water.

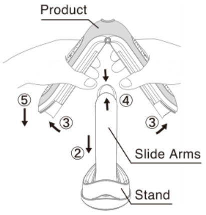

①Towel Dry
②Slot Slide Arms into Stand
③Hold FLIP ZERO with both hands
④Pinch Slide Arms with thumb and middle finger
⑤Place FLIP ZERO on to Slide Arms

How to Store

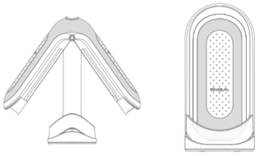

①Ensure product is fully dried.
②Place closed product onto Stand and cover with Clear Cover.
③Store in a cool, dry, dark place away from direct sunlight.

Warning

Male Pleasure Item
Do not use on irritated or damaged skin.
Spot test on base of arm first to ensure no irritation occurs on skin contact. Contact medical professional should irritation occur.
Ensure product is fully washed and dried after use and before storage.
Use at room temperature.
Keep out of reach of children.
TENGA Co., Ltd. can not be held resposible if any parts are accidentally injested.
Do not intentionally damage product.
Do not share.
This is a novelty item. TENGA Co., Ltd. can not be held responsible for any other use than its intended use.
Use at own discretion.
Reusable. Do not use if damaged.

TENGA FLIP 0 (ZERO)

TFZ-001

Materials: Elastomer / PC / GPPS / ABS / Silicone
Product Size 180×80×70mm / 480g

For further information visit: www.tenga-global.com

U.S. Patent 8,353,887 / Europe Patent 2168500 / Japan Patent 5261382
Design Patent Pending

Manufactured by TENGA Co., Ltd.

[TFZ01P02-10]

Annex 3

.

This first week of treatment will start with an exercise that intends to raise awareness of the ejaculatory response, for which we will use this Japanese device called Flip Zero.

TARGET

1. We intend to provide a better understanding of the role of pelvic floor in the ejaculatory response .
2. Pay attention to see what happens to the muscles of the genital area a minute or two before ejaculating.
3. Notice how the muscles in the genital area contract. This muscular tension in the area of what we call pelvic floor is necessary to ejaculate.

KEYS

The activity realizing only once seen the video corresponding to the activity Discovering the Pelvic Floor.

The activity is realized individually.

The man must deal before the activity realizes the theoretical content of the same one.

There will be realized a minimum of 4 times or until the man is capable of reaching the aims of this first week.

EXERCISE

This first week of treatment will start with an exercise that intends to raise awareness of the ejaculatory response, for which we will use this Japanese device called Flip Zero.

To open it, remove this piece and press the middle on both sides. Once opened, apply lubricant on the inside, and then press to close it and insert this piece. Now you need to use lubricant for the entry. That’s it, ready to use.

Masturbate with the device as you normally do and pay attention to see what happens to the muscles of the genital area a minute or two before ejaculating.

Notice how the muscles in the genital area contract, especially in the anal area and under the testes. This muscular tension in the area of what we call pelvic floor is necessary to ejaculate.

.

For the next three weeks, do an exercise that helps to learn to voluntarily control the muscles of the genital area during masturbation. To do so, continue using the device.

TARGET

1. To know the physiological changes of a sexual masculine complete cycle.
2. To identify in the body the changes in the musculature of the genital area of the phase pre-orgasmic
3. To identify the voluntary character of the muscular tension that initiates the phase pre-orgasmic
4. To start developing control on this musculature involved in the phase pre-orgasmic.

KEYS

To realize the activity only once to have identified in the activity the muscular tension in the genital zone before the ejaculation and to understand therefore the role that plays the pelvic floor in the orgasmic response

The activity realizes individually, trying that her duration is not lower than 5 minutes.

The man must deal before the activity realizes the theoretical content of the same one.

It will be realized so often since like be necessary until the man is capable of reaching the aims of this second exercise, there is recommended a minimum of 4 times for week.

EXERCISE

Masturbate as you normally do, but this time stop approximately one minute before ejaculating.

With the penis inside the device, identify the tension on the muscles of the genital area and try to reduce this tension, try to relax the pelvic floor muscles pay special attention to the anal sphincter. This stop should not last more than a minute.

If you've managed to loosen muscle tension in the area, continue masturbating. Again, about a minute before you ejaculate, stop and try to loosen the tension in the area. These stops should not last more than a minute.

Try to make at least four stops before ejaculating. You should do this activity four times a week for the next three weeks.

For the next two weeks, do an exercise that helps to learn to voluntarily control the muscles of the genital area during masturbation. To do so, continue using the device.

OBJETIVOS

1. To know the physiological changes of a sexual masculine complete cycle.

2. To identify in the body the changes in the musculature of the genital area of the phase pre-orgasmic

3. To identify the voluntary character of the muscular tension that initiates the phase pre-orgasmic

4. To guarantee the control of the phase pre-orgasmic across the musculature of the pelvic floor in conditions similar to the coitus.

KEYS

To realize the activity only once to have identified in the activity the muscular tension in the genital zone before the ejaculation and to understand therefore the role that plays the pelvic floor in the orgasmic response

The activity realizes individually, trying that her duration is not lower than 5 minutes.

The man must deal before the activity realizes the theoretical content of the same one.

It will be realized so often since like be necessary until the man is capable of reaching the aims of this exercise, there is recommended a minimum of 4 times for week.

EXERCISE

In the next activity we will also make stops when we feel that we have about a minute to ejaculate but this time we will not stop masturbating. Focus on loosening tension in the genital area as in the previous exercise, pay special attention on the pelvic floor and particularly in the anal sphincter but at no time will we cease stimulation.

You must therefore try to delay the ejaculatory response on four different occasions, by loosening the tension in the genital area and relaxing the anal sphincter. You can ejaculate once you have made these stops while stimulating.

Try to do this activity 4 times a week for two weeks.

In the last activity you will practice the ability to delay ejaculation as in previous exercises, but this time performing hip movements similar to penetration.

TARGET

1. To know the physiological changes of a sexual masculine complete cycle.

2. To identify in the body the changes in the musculature of the genital area of the phase pre-orgasmic

3. To identify the voluntary character of the muscular tension that initiates the phase pre-orgasmic

4. To guarantee the control of the phase pre-orgasmic across the musculature of the pelvic floor in conditions similar to the coitus with movements of hip .

KEYS

To realize the activity only once to have identified in the activity the muscular tension in the genital zone before the ejaculation and to understand therefore the role that plays the pelvic floor in the orgasmic response

The activity realizes individually, trying that her duration is not lower than 5 minutes.

The man must deal before the activity realizes the theoretical content of the same one.

It will be realized so often since like be necessary until the man is capable of reaching the aims of this last exercise, there is recommended a minimum of 4 times for week.

EXERCISE

In the last activity you will practice the ability to delay ejaculation as in previous exercises, but this time performing hip movements similar to penetration.

While standing, hold the device against a surface and move your hips as if you were performing a penetration.

Try to delay the ejaculatory response four times, loosening muscle tension in the genital area while moving your hips. Remember to relax your anal sphincter to delay ejaculation.

Try to do this activity 4 times a week for one week.
