# Supplementary material for: Efficacy of Sphincter Control Training (SCT) in the treatment of premature ejaculation, a new cognitive behavioral approach: A parallel-group randomized, controlled trial
Source: PLoS One. 2019 Feb 26;14(2):e0212274. doi: 10.1371/journal.pone.0212274 (PMC6391003; doi:10.1371/journal.pone.0212274)
Supplement: S4 File — Study protocol Trial ISM-FLI-2017-01 in spanish. (PDF) [file pone.0212274.s004.pdf]

1. IDENTIFICACION DEL PROTOCOLO

Código del estudio :
ISM-FLI-2017-01

2. TITULO DEL PROTOCOLO

PROTOCOLO DE INVESTIGACION FLIP ZERO
(Tratamiento de la Eyaculación Precoz Primaria)

3. IDENTIFICACION DEL INVESTIGADOR

INVESTIGADORES: .

Sr. Jesús Eugenio Rodríguez Martínez
Licenciatura en Psicología.
Master Psicología Clínica.
Master Sexología y Sexualidad Humana.
Doctorando Departamento Psicologia de la Salud UMH.
Director Instituto Sexológico Murciano (ISM).

Sr. Jose Antonio Picazo Aroca.
Licenciatura en Psicología.
Máster en Neurociencias Básicas y Aplicadas.
Doctorando Departamentos Psicologia de la Salud UMU.
Area de investigación de Instituto Sexológico Murciano (ISM).

Dr. José Antº Piqueras Rodríguez.
Profesor Titular.
Área de Personalidad, Evaluación y Tratamiento Psicológico
Departamento de Psicología de la Salud.
Universidad Miguel Hernández de Elche (UMH).

Dr. Juan Carlos Marzo Campos.
Profesor Titular.
Área de Psicología Social.
Director Departamento de Psicología de la Salud.
Universidad Miguel Hernández de Elche (UMH).

4. PROMOTOR

Sr. Jesús Eugenio Rodríguez Martínez
Insituto Sexologico Murciano
C/ Periodista Encarna Sanchez 22,
30007, Murcia

5. RESUMEN

5.1. Identificación del promotor

Instituto Sexológico Murciano (ISM)
C/Periodista Encarna Sanchez ,22, P1 30007 Murcia
Teléfono: 868 94 14 18
N.I.F 48478368A

5.2. Título del ensayo clínico

Estudio aleatorizado para comparar la eficacia y seguridad de un protocolo de ejercicios que utiliza un dispositivo de ayuda a la masturbación como tratamiento de primera linea para pacientes con eyaculación precoz primaria.

5.3. Código del protocolo

Código del protocolor: ISM-FLI-2017-01

5.4. Investigador principal.

Sr. Jesús Eugenio Rodríguez Martínez
Director Insituto Sexologico Murciano
C/ Periodista Encarna Sanchez 22,
30007, Murcia

5.5. **Centros en los que se prevé realiza**r el estudio .

El estudio se prevé desarrollarlo en el centro sanitario siguiente:

1. Instituto Sexológico Murciano
C/ Periodista Encarna Sanchez Nº 22, P1 Nº 2.
30007 Murcia. Tel. 868 94 14 18
RES: 40000319

5.6. CEIC de referencia y centros participantes.

Este ensayo ha sido sometido a la aprobación del comité ético de referencia siguiente:

- Hospital José Mª Morales Meseguer – Murcia

En este estudio participarán los centros siguientes:

-Instituto Sexológico Murciano-Murcia

5.7. Objetivo principal

Comparar la eficacia y seguridad del uso de un nuevo programa de ejercicios con el que se utiliza un dispositivo de ayuda a la masturbación en el tratamiento de la eyaculación precoz primaria en población española.

5.8. Diseño

Este proyecto de investigación utilizará dos grupos experimentales, a uno se le administró la terapia “star-stop” (experimental 1) y otro la terapia “star-stop” combinada con el dispositivo externo (experimental 2). Los sujetos de ambos grupos recibiran una terapia de forma paralela y con el mismo protocolo de ejercicios (con la única diferencia de que en el experimental 2 los ejercicios de parada y arranque se haran con el dispositivo externo). La asignación a cada una de las condiciones sera aleatoria por orden de entrada al estudio de aquellos que cumplían con los criterios de selección.

5.9. Enfermedad o trastorno en estudio

Eyaculación Precoz Primaria (F52.4).

5.10. *Datos sobre el dispositivo objeto de estudio y el programa de ejercicios* **:**

Dispostivo objeto del estudio: Flip Zero.

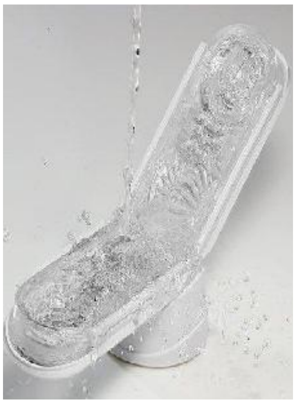

Flip Zero TFZ-001 , TENGA Co., Ltd.

Dispositivo de ayuda a la masturbación hipoalergénico y con tratamiento antibacteriano reutilizable.

Tamaño: 70×80×180mm / (D)×(W)×(H)
Tamaño embalado: 77×92×213mm / (D)×(W)×(H)
Peso embalado: 460g
Otros datos: reutilizable + 100 usos.
Manual de Instrucciones ANEXO 2

Programa de ejercicios : Star-Stop 3.0.

El programa de ejercicios para realizar en casa consta de 4 actividades:

El primer ejercicio denominado "Descubriendo el suelo pélvico se realizará durante una semana, el segundo se denominada "Parada y Arranque" y debía realizarse durante 3 semanas, el tercero denominado "Parada y Arranque sin cese de estimulación" se realizaba durante 2 semanas y el última ejercicio del programa se denominaba "Parada y Arranque sin cese de la estimulación con movimientos coitales" que se desarrollaba durante una semana.
Fichas ejercicios ANEXO 3

Lubricante usado para el estudio: HOLE LOTION REAL.

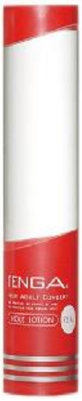

HOLE LOTION [REAL]: TLH-002 de TENGA Co., Ltd.

Tamaño embalado: 44×44×177mm / (D)×(W)×(H)
Volumen 170ml.

Ingredientes: agua, glicerina, etanol, poliacrilato de sodio, hidroxicelulosa, fenoxietanol, EDTA-2Na, parabeno, polisorbato 80, cocoato de sorbitán, fragancia

5.11. Población en estudio y número total de sujetos.

La población en estudio serán hombres heterosexuales de 18 años o más de edad, de todo el territorio nacional, que padezcan eyaculacion precoz primaria, que no hayan recibido ningún tratamiento previo y que tengan pareja estable al menos 6 meses antes de comenzar el ensayo.

Se prevé un tamaño muestral de 50 pacientes. Se calcula un 5% de abandonos o incumplimiento durante el estudio.

5.12 Calendarioy fecha prevista de finalización

Duración periodo de inclusión:Octubre 2016 – Junio 2017.
Fecha prevista inicio del estudio: Julio 2017
Fecha prevista final del estudio: Septiembre 2017

5.13. Fuente de financiación

La financiación correra a cargo del área de investigación del Instituto Sexológico Murciano y de la multinacional japonesa TENGA Co .

6. ASPECTOS RELEVANTES SOBRE LA FINANCIACION DEL ESTUDIO

El 70 % de la financiación del proyecto correrá a cargo del área de investigación del Instituto Sexológico Murciano ,mientras que el otro 30% sera costeado por parte de la compañia japonesa TENGA Co.

Este 30 % incluye la cesión de de 30 dispositivos Flip Zero TFZ-001 y de 30 botes de lubricante HOLE LOTION [REAL]: TLH-002 asi como la producción y grabación de un video con los distintos ejercicios del programa de tratamiento.

MEMORIA ECONÓMICA
Código del estudio: ISM-FLI-2017-01

MEMORIA ECONÓMICA DE LOS GASTOS PREVISTOS DEL PROYECTO DE INVESTIGACIÓN:

Título: **PROTOCOLO DE INVESTIGACION FLIP ZERO**
Código del estudio: **ISM-FLI-2017-01**

Investigador/a Principal: **Jesús Eugenio Rodríguez Martínez**
Promotor: **Instituto Sexológico Murciano**

DESCRIPCIÓN DETALLADA DE LOS GASTOS PREVISTOS
CON CARGO AL PROYECTO

**GASTOS DE PERSONAL:**	
1 Becario área de investigación media jornada	400,00 €/mes * 3
SUBTOTAL PAGOS A PERSONAS:	1.200 ,00 €
**INSTALACIONES Y MATERIALES, LICENCIAS DE SOFTWARE Y TEST:**	
Licencias y kit de corrección MCMI-III , PEP, PEDT y GRISS male.<br>1 Licencia mensual IBM spss stadistics software.<br>Envio agencia MRW ecommerce nacional transporte 30 kit tratamiento.<br>Grabación, edición y producción video ejercicios Conrad Son Media.	66,49 €<br>88,50 €<br>275,10 €<br>1.800,00 €
SUBTOTAL INSTALACIONES Y EQUIPOS:	2.230,9 €
**MATERIAL FUNGIBLE:**	
Impuestos Aduana 30 KIT (HOLE LOTION [REAL]: TLH-002 y Flip Zero TFZ-001)<br>Valor declarado 30 KIT (HOLE LOTION + FLIP ZERO)	27,51 €<br>131,02 €
SUBTOTAL MATERIAL FUNGIBLE:	158,53 €

1

MEMORIA ECONÓMICA
Código del estudio: ISM-FLI-2017-01

**OTROS GASTOS :** (especificar con detalle la naturaleza del gasto y cantidades abonadas)	
Campaña marketing sanitario captación de sujetos (Anuncios y buscadores) Flint Spain S.L.	1.875,50 €
Dominio y hosting, web de aterrizaje estudio, yocontrolo.org , Arsys S.L.U.	101,41€
Plantilla web .Web Templates Ltd	67,00 €
Tasas CEIC Morales Meseguer	669,63 €
SUBTOTAL OTROS GASTOS:	2.713,54 €
**TOTAL PRESUPUESTO DEL PROYECTO:**	**6.302,97 €**

7. CENTRO DONDE SE VA A DESARROLLAR EL ENSAYO

El estudio se va a desarrollar en el Instituto Sexológico Murciano (ISM) centro sanitario privado.
Tipo de centro y Código: C.2.2 Consulta de otros profesionales sanitarios.
Servicios autorizados y código: U.900.1 OTRAS UNID.ASIST.PSICOLOGIA
Domicilio: Calle Periodista Encarna Sanchez . Planta 1 , nº 2 , 22, Murcia.
Autorización de funcionamiento: 09/10/2013
Inscripción en el Registro de la Conserjeria de Sanidad con el Nº: 40000319

8. JUSTIFICACION Y PERTINENCIA DEL ENSAYO

La eyaculación precoz (EP) entendida como una condición en la que se da una falta de control eyaculatorio acompañado de tiempos de latencia eyaculatoria intravaginal (TLEI) cortos (de hasta un minuto), con una estimulación mínima y antes de que la persona lo desee (Serefoglu et al., 2014) es considerada la disfunción sexual masculina más frecuente, ya que afecta a millones de hombres en todo el mundo (Russo et al., 2016). En la actualidad los tratamientos para la EP consisten en una combinación de fármacos y terapias sexológicas o psicológicas, en especial técnicas conductuales para el hombre y su pareja.
Las teorías psicológicas intentan explicar la etiología de la EP desde un punto de vista que incluye el efecto de la experiencia temprana y el condicionamiento sexual, la ansiedad, la técnica sexual, la frecuencia de la actividad sexual, y explicaciones psicodinámicas del desarrollo (p.e, Brody & Weiss, 2015). Sin embargo, son las teorías biológicas las que poseen más evidencia científica (p.e, Waldinger, 2002, Corona et al., 2008). De entre estas, el modelo clásico explica el proceso de eyaculación humano en el que la emisión, con la formación de una “cámara de presión” (creada en la uretra prostática), es seguida de la expulsión (contracciones rítmicas del músculo bulboesponjoso) del fluido seminal (Marberger, 1974).
Aunque las nuevas y, a menudo mejor vendidas terapias farmacológicas están eclipsando los métodos psicológicos y conductuales tradicionales en el tratamiento de la EP, a día de hoy no hay evidencia de que en la base de la EP sólo haya una causa fisiológica (Althof et al., 2014). Además, se ha comprobado que las estrategias psicológicas, basadas en técnica conductuales, para el tratamiento de la EP han tenido cierto éxito en el alivio de esta disfunción (Althof et al., 2006).
Sólo unos pocos estudios han comparado las técnicas psicoterapéuticas para la EP, ya sea frente a una condición de control (que queda en lista de espera) o contra otro tratamiento psicológico. La falta de protocolos de tratamiento específicos y de fondos de investigación para llevar a cabo estudios de comprobación bien diseñados de estos protocolos ha disminuido el atractivo de estos enfoques en relación a la evolución de las estrategias farmacológicas.

Esto a pesar de que varias estrategias cognitivo-conductuales se acercan al cumplimiento de los criterios de apoyo empírico.
Entre ellas, el método de "start-stop", desarrollado por Semans (1956) y adaptado por otros autores como Glina et al (2007), suprime la urgencia de eyacular deteniendo o pausando la estimulación sexual antes de llegar al punto de inminente eyaculación mediante un apretón del glande. Además, se sabe que el uso de dispositivos externos puede resultar eficaz para ayudar a controlar la eyaculación (Rodríguez & López, 2016).
Este proyecto pretende demostrar la eficacia sobre la EP del tratamiento combinado “star-stop” adaptado de Semans (1956) en el marco de un programa de ejercicios llevados a cabo con un dispositivo externo similar al empleado en Rodríguez & López (2016). El estudio pretende aportar evidencia científica y metodológica a un tratamiento cognitivo-conductual ofreciendo una alternativa a los tratamientos farmacológicos actuales para la EP.

9. DISEÑO Y JUSTIFICACION

Este proyecto de investigación utilizará dos grupos experimentales, a uno se le administrará la terapia “star-stop” (experimental 1) y otro la terapia “star-stop” combinada con el dispositivo externo (experimental 2). Los dos grupos recibirán terapia de forma paralela y con el mismo protocolo de ejercicios (con la única diferencia de que en el experimental 2 los ejercicios de parada y arranque se harán con el dispositivo externo). La asignación a cada una de las condiciones será aleatoria por orden de entrada al estudio de aquellos que cumplían con los criterios de selección.

En ambos grupos, y para permitir que los dos grupos de sujetos puedan acceder al tratamiento para la EP, el control es intra-sujetos. Se recogeran, durante un periodo similar al de tratamiento, medidas de las principales variables que serán utilizadas para establecer la línea base de cada grupo.

10. OBJETIVO PRINCIPAL

El objetivo del proyecto es demostrar la eficacia sobre la EP del tratamiento combinado “star-stop” adaptado de Semans (1956) en el marco de un programa de ejercicios llevados a cabo con un dispositivo externo similar al empleado en Rodríguez & López (2016).
La eficacia sobre la EP se verá reflejada en un aumento en los tiempos de latencia de eyaculación intravaginal (TLEI) y en puntuaciones de percepción subjetiva, de la terapia sobre la línea base. La mayor eficacia del tratamiento combinado (“star-stop” junto con dispositivo externo) se verá reflejada en el mayor aumento de estos TLEI y en puntuaciones de percepción subjetiva de la terapia combinada sobre la terapia “star-stop”.
El nivel de evidencia científica marcará la competitividad de esta técnica psicológica cognitivo-conductual

11. VARIABLE PRINCIPAL DE VALORACION

La principal variable de medida es el tiempo de latencia de eyaculación intravaginal (TLEI). Esta es una variable que calcula el propio sujeto experimental y es el tiempo, en segundos, desde que comienza la penetración hasta que eyacula. Se trata de la variable más estudiada en investigaciones de este tipo (Waldinger, Zwinderman, Olivier, & Schweitzer, 2008).

Otras variables que se tienen en cuenta son de naturaleza más psicólogica y de carácter autoinformado por los propios pacientes: Por un lado, la percepción que tienen los sujetos sobre su propia eyaculación: el estrés personal que les provoca, la satisfacción con las relaciones sexuales y la dificultad interpersonal relacionada con la eyaculación y, por otro, variables relacionadas con la satisfacción sexual como la percepción de impotencia, percepción de eyaculación precoz, evitación de las relaciones sexuales, falta de sensualidad y nivel de satisfacción/insatisfacción con la relaciones sexuales.

12. POBLACIÓN EN ESTUDIO Y NÚMERO TOTAL DE PACIENTES

Los sujetos que se tendrán en cuenta serán todos los interesados en participar que respondan a la campaña de publicidad a nivel nacional que se pondrá en marcha.Estos serán contactados por correo electrónico o vía telefónica, los cuestionarios y registros de selección serán enviados por correo electrónico (el criterio de inclusión en el estudio se define en el siguiente apartado).

Tambien podrán participar en el estudio todos aquellos pacientes que acudan al ISM solicitando evaluación y tratamiento para la EP y que cumplan los criterios de inclusión. Los sujetos serán libres de abandonar el estudio en cualquier momento y no recibirán compensación económica por participar, aunque recibirán un regalo a la finalización del estudio , se prevé una tasa elevada de abandonos .

En base a estudios randomizados para valorar la eficacia de tratamientos para la eyaculación precoz en los que existía grupo de control, estimamos que será necesario 24 sujetos por grupo para establecer diferencias considerando una potencia estadística de 0.80 y un alpha de p=0.05.

13. CRITERIOS DE INCLUSION Y DE EXCLUSION DE PACIENTES

Criterios de inclusión para ser seleccionado serán: Tener más de 18 años, estar en una relación heterosexual al menos durante los últimos 6 meses, tener una puntuación mayor que 11 en el PEDT (Premature Ejaculation Diagnostic Tool) y un TLEI (Tiempo de latencia eyaculatoria intravaginal) medio autoinformado <120 segundos.

Los criterios de exclusión incluirán: Historial de abuso o dependencia de alcohol, haber recibido mediación o tratamiento psicológico para la EP en los últimos 6 meses, padecer diabetes o el uso habitual de drogas recreativas (exceptuando tabaco y cafeína).

14. ANALISIS ESTADISTICO

Como medida principal se utilizará el "fold increase" de los TLEI que se calculará usando las medias geométricas de los TLEI post- tratamiento (periodo B) dividida por la media de los TLEI al empezar el tratamiento que servían de línea base (periodo A).
Se utilizará la prueba de la distancia de Mahalanobis para la comprobación de existencia de Outliers (en los que la puntuación Z > 3) y Kolmogorov-Smirnov para comprobar la bondad de ajuste a la normalidad de los datos y la transformada sqrt en aquellos casos en los que no se cumpla el supuesto de normalidad.
Se utilizará el método de Greenhouse-Geisser para corregir los grados de libertad cuando no se cumpla el supuesto de esfericidad.
Se realizará el contraste MANOVA para las variables demográficas, presumiblemente encontraremos diferencias significativas en variables como edad
Para los contrastes se utilizarán ANCOVAS de medidas independientes, con las variables que hayan salido significativas en el contraste MANOVA como covariada, para comprobar las diferencias entre los dos grupos experimentales y la igualdad entre líneas base en cada una de las variables de estudio. Y, de forma similar, ANCOVAS de medidas repetidas para comprobar la existencia de los beneficios de cada terapia sobre la línea base.
Todos los valores de p deberán de ser bilaterales y el nivel de significación utilizado < 0.05. Se utilizará el paquete estadístico IBM SPSS Statitisc versión 22.

15. CONSIDERACIONES ETICAS

Este estudio deberá desarrollarse de acuerdo con el protocolo y con las normas de Buena Práctica Clínica (GCP), tal como se describe en: Normas Tripartitas Armonizadas de la ICH para Buena Práctica clínica 1996.
El investigador accede, con la firma de este protocolo, a seguir las instrucciones y procedimiento descritos en el mismo y por lo tanto cumplirá los principios de Buena Práctica Clínica en los cuales se basa teniendo presente la Declaración de Helsinki http://www.unav.es/cdb/ammhelsinki2.html.
Todos los pacientes pasarán por una condición terapéutica, en el caso de la terapia "star-stop" la eficacia está comprobada por estudios anteriores (Glina et al., 2007) y en el caso de la terapia combinada, el riesgo para la salud es mínimo y la probabilidad de su eficacia es como mínimo igual que la terapia "star-stop" ya que, en este caso, la primera engloba a la segunda.
Además, a la terminación del estudio se concederá un regalo para aquellos que lo concluyan valorado entre 17 y 92€ en concepto de "bote de 170 ml de lubricante" y "dispositivo de ayuda a la masturbación".
Los datos recogidos estarán sujetos a la ley de protección de datos: Ley orgánica 15/1999 de 13 de diciembre de Protección de Datos de Carácter Personal (LOPD). Y el consentimiento informado se recoge de conformidad con el Real Decreto 223/2004 de 6 de febrero.

16. DURACION DEL TRATAMIENTO

La duración del tratamiento será de 7 semanas durante las cuales se practicarán una serie de ejercicios anteriormente explicados.

17. EVALUACION Y SEGURIDAD DEL ESTUDIO

El material empleado en la investigación será inocuo y su uso responsable no produce reacciones adversas de interés.

Se procederá a la creación de un fichero de datos de carácter personal con titularidad de Instituto Sexológico Murciano, con la finalidad de la recogida de éstos y estudio posterior.

Se conservará una copia de respaldo de los datos y de los procedimientos de recuperación de los mismos en un lugar diferente de aquel en que se encuentren los equipos informáticos que los tratan

Asignación a cada sujeto un código numérico o alfanumérico según un esquema de aleatorización generado estadísticamente.

El responsable del fichero o del tratamiento garantiza que únicamente él tiene acceso y trata los datos personales.

Los datos confidenciales se cifrarán con un algoritmo de cifrado y una clave que los hace ilegibles si no se conoce dicha clave.

Los ficheros no automatizados se almacenarán en armarios o archivadores de documentos, que a su vez, en un lugar con acceso protegido mediante puertas con llave.

El lubricantes usado esta fabricado con ingredientes inocuos. No debería haber problemas en caso de ingestión. No obstante, en caso de irritación dejarán de usarlo y serán excluido de la investigación. En caso de alergias los usuarios serán informados de los ingredientes previos a su utilización y a todos se les indicara que hagan un pequeño test de tolerancia en el brazo antes de usarlo en la zona genital .

18. CALENDARIO Y FECHA PREVISTA DE FINALIZACION

El periodo de selección e inclusión de pacientes para participar en el estudio se fija entre Octubre 2016 y Junio 2017.

La fecha prevista de inicio del estudio e inicio de los tratamientos es Julio 2017

La fecha prevista para finalizar con el programa de ejercicios en ambos grupos se estima a finales de Septiembre 2017.

19. ENCUESTAS, ENTREVISTAS O INSTRUMENTOS QUE SE UTILICEN EN EL ESTUDIO

La medida de TLEI se recogerá a través de un autorregistro por semana de tratamiento (ANEXO I).

Para la recogida de información relativa a la percepción sobre la eyaculación, se utilizará el Premature Ejaculation Profile (PEP) (Patrick et al., 2009), un instrumento autoinformado que consta de cuatro medidas (Control percibido sobre la eyaculación, estrés personal relacionado con la eyaculación, satisfacción con las relaciones sexuales y dificultad interpersonal relacionada con la eyaculación) que arrojan un perfil y una puntuación general (Patrick et al., 2009) donde las peores puntuaciones expresan un peor rendimiento. El coeficiente de fiabilidad entre clases va del 0.66 al 0.83 y su validez fue contrastada con la dapoxetina y los Tiempos de Latencia de Eyaculación Intravaginal (TLEI). Este instrumento fue validado sobre población estadounidense y europea con alemanes, francés, ingleses, italianos y polacos (Giuliano et al., 2008) aunque se ha utilizado también en población española (Bar-Or, Salottolo, Orlando, Winkler, & Group, 2012; Buvat, Tesfaye, Rothman, Rivas, & Giuliano, 2009).
Para el diagnóstico se usará el Premature Ejaculation Diagnostic Tool (PEDT) que mide cinco ítems que evalúan: dificultad para retardar la eyaculación, eyaculación antes que el paciente lo desea, eyaculación con poca estimulación, frustración relacionada al eyacular prematuramente y opinión de la pareja sobre la eyaculación. . La fiabilidad test-retest es de 0.82 y todos los ítems discriminan de forma estadísticamente significativa entre pacientes con PE y sin PE.

Tambien se administará el Inventario de Satisfacción Sexual de Golombok y Rust (GRISS, siglas en inglés). Se trata de un breve cuestionario de 28 preguntas para evaluar la existencia y gravedad de los problemas sexuales de cada uno de los miembros de las parejas heterosexuales (Rust & Golombok, 1985, 1986). Este cuestionario proporciona puntuaciones para hombres y mujeres por separado en 3 escalas compartidas (evitación, no sensualidad e insatisfacción) y otras 6 específicas de cada sexo (Impotencia, eyaculación precoz, anorgasmia, vaginismo, infrecuencia y falta de comunicación) con unos valores aceptables de fiabilidad y validez (Rust & Golombok, 1985, 1986). El instrumento fue validado en población inglesa y ya se comienza a utilizar en algunos trabajos españoles del máster oficial de Sexología de la universidad de Almería (García González, 2013; López Lorente, 2013).

El dispositivo externo de ayuda a la masturbación será el Flip-Hole de la compañía TENGA, Co .

Flip Zero TFZ-001 , TENGA Co., Ltd.
Dispositivo de ayuda a la masturbación hipoalergénico y con tratamiento antibacteriano reutilizable.

Tamaño: 70×80×180mm / (D)×(W)×(H)
Tamaño embalado: 77×92×213mm / (D)×(W)×(H) Peso embalado: 460g
Otros datos: reutilizable + 100 usos.

El lubricante usado durante el estudio se denomina:

HOLE LOTION [REAL]: TLH-002 de TENGA Co., Ltd.

Volumen 170ml.

Tamaño embalado: 44×44×177mm / (D)×(W)×(H)

Ingredientes: agua, glicerina, etanol, poliacrilato de sodio, hidroxicelulosa, fenoxietanol, EDTA-2Na, parabeno, polisorbato 80, cocoato de sorbitán, fragancia

20. REFERENCIAS BIBLIOGRAFICAS

Althof, S. E., McMahon, C. G., Waldinger, M. D., Serefoglu, E. C., Shindel, A. W., Adaikan, P. G., … Torres, L. O. (2014). An Update of the International Society of Sexual Medicine's Guidelines for the Diagnosis and Treatment of Premature Ejaculation (PE). Sexual Medicine, 2(2), 60–90. https://doi.org/http://dx.doi.org/10.1002/sm2.28

Althof, S., Rosen, R., Symonds, T., Mundayat, R., May, K., & Abraham, L. (2006). Development and validation of a new questionnaire to assess sexual satisfaction, control, and distress associated with premature ejaculation. The Journal of Sexual Medicine, 3(3), 465–475.

Bar-Or, D., Salottolo, K. M., Orlando, A., Winkler, J. V, & Group, T. O. D. T. S. (2012). A randomized double-blind, placebo-controlled multicenter study to evaluate the efficacy and safety of two doses of the tramadol orally disintegrating tablet for the treatment of premature ejaculation within less than 2 minutes. European Urology, 61(4), 736–743.

Brody, S., & Weiss, P. (2015). Erectile Dysfunction and Premature Ejaculation: Interrelationships and Psychosexual Factors. The Journal of Sexual Medicine, 12(2), 398–404. https://doi.org/http://dx.doi.org/10.1111/jsm.12738

Buvat, J., Tesfaye, F., Rothman, M., Rivas, D. A., & Giuliano, F. (2009). Dapoxetine for the Treatment of Premature Ejaculation: Results from a Randomized, Double-Blind, Placebo-Controlled Phase 3 Trial in 22 Countries. European Urology, 55(4), 957–968. https://doi.org/10.1016/j.eururo.2009.01.025

Corona, G., Jannini, E. A., Mannucci, E., Fisher, A. D., Lotti, F., Petrone, L., … Forti, G. (2008). Different testosterone levels are associated with ejaculatory dysfunction. The Journal of Sexual Medicine, 5(8), 1991–1998.

García González, S. (2013). Deseo, autoestimulación, satisfacción y fantasías sexuales en personas con necesidades especiales. Universidad de Almería. Retrieved from http://repositorio.ual.es/bitstream/handle/10835/2542/Trabajo.pdf?sequence=6

Giuliano, F., Patrick, D. L., Porst, H., La Pera, G., Kokoszka, A., Merchant, S., … Polverejan, E. (2008). Premature Ejaculation: Results from a Five-Country European Observational Study. European Urology, 53(5), 1048–1057. https://doi.org/10.1016/j.eururo.2007.10.015

Glina, S., Abdo, C. H., Waldinger, M. D., Althof, S. E., Mc Mahon, C., Salonia, A., & Donatucci, C. (2007). Premature ejaculation: A new approach by James H. Semans. Journal of Sexual Medicine, 4(4), 831.

López Lorente, A. M. (2013). Las relaciones de apego y su influencia en la satisfacción sexual de enfermos/as alcohólicos/as. Universidad de Almería. Retrieved from http://repositorio.ual.es/bitstream/handle/10835/2365/Trabajo.pdf?sequence=1&isAllowed=y

Marberger, H. (1974). The mechanisms of ejaculation. In Physiology and genetics of reproduction (pp. 99–110). Springer.

Patrick, D. L., Giuliano, F., Ho, K. F., Gagnon, D. D., McNulty, P., & Rothman, M. (2009). The Premature Ejaculation Profile: validation of self-reported outcome measures for research and practice. BJU International, 103(3), 358–364. https://doi.org/10.1111/j.1464-410X.2008.08041.x

Rodríguez, J. E., & López, A. (2016). Male masturbation device for the treatment of premature ejaculation. Asian Pacific Journal of Reproduction, 5(1), 80–83.
Russo, A., Capogrosso, P., Ventimiglia, E., La Croce, G., Boeri, L., Montorsi, F., & Salonia, A. (2016). Efficacy and safety of dapoxetine in treatment of premature ejaculation: an evidence□based review. International Journal of Clinical Practice, 70(9), 723–733.
Rust, J., & Golombok, S. (1985). The Golombok-Rust Inventory of Sexual Satisfaction (GRISS). British Journal of Clinical Psychology, 24(1), 63–64. https://doi.org/10.1111/j.2044-8260.1985.tb01314.x
Rust, J., & Golombok, S. (1986). The GRISS: a psychometric instrument for the assessment of sexual dysfunction. Archives of Sexual Behavior, 15(2), 157–165.
Semans, J. H. (1956). Premature ejaculation: A new approach. Southern Medical Journal, 49(4), 353–358.
Serefoglu, E. C., McMahon, C. G., Waldinger, M. D., Althof, S. E., Shindel, A., Adaikan, G., … Torres, L. O. (2014). An Evidence-Based Unified Definition of Lifelong and Acquired Premature Ejaculation: Report of the Second International Society for Sexual Medicine Ad Hoc Committee for the Definition of Premature Ejaculation. Sexual Medicine, 2(2), 41–59. https://doi.org/http://dx.doi.org/10.1002/sm2.27
Waldinger, M. D. (2002). The neurobiological approach to premature ejaculation. The Journal of Urology, 168(6), 2359–2367.
Waldinger, M. D., Zwinderman, A. H., Olivier, B., & Schweitzer, D. H. (2008). Geometric mean IELT and premature ejaculation: appropriate statistics to avoid overestimation of treatment efficacy. The Journal of Sexual Medicine, 5(2), 492–499

Firma promotor:
Instituto Sexológico Murciano

INSTITUTO SEXOLOGICO MURCIANO
N.R.S 20300055
Tel [illegible]
[illegible]

Firma investigador Principal:
D. Jesús Eugenio Rodríguez Martínez

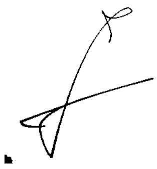

ANEXO 1

Cuaderno de recogida de datos

REGISTROS TIEMPO DE EYACULACION

SEMANA:

NOMBRE:

Instituto Sexológico Murciano

	LUNES	MARTES	MIERCOLES	JUEVES	VIERNES	SABADO	DOMINGO
**MASTURBACION** — Indique el **tiempo que tarda en eyacular** cuando se **masturba**							
**PENETRACION COITO** — Indique el **tiempo que tarda en eyacular** despues de **penetrar**							

Autoregistro de la latencia eyaculatoria
(Rodriguez & López, 2014)

ANEXO 2

Manual Instrucciones dispositivo

FLIP ZERO 0

Manual de usuario

Spanish

De cero al infinito: puro placer

Con los materiales de mayor calidad y la tecnología más moderna, TENGA presenta lo último en tecnología para el placer masculino: FLIP ZERO.

¡La exclusiva serie FLIP de TENGA evoluciona y abre nuevos horizontes de placer!

Su punto de inserción sin juntas evita las fugas de lubricante y permite la formación de un potente vacío mediante una válvula unidireccional.

FLIP 0 (ZERO) presenta los detalles internos más intrincados nunca vistos para lograr la máxima sensación. Además, nuestra pionera función abrefácil FLIP permite una perfecta limpieza para poder reutilizar el producto.

Partes

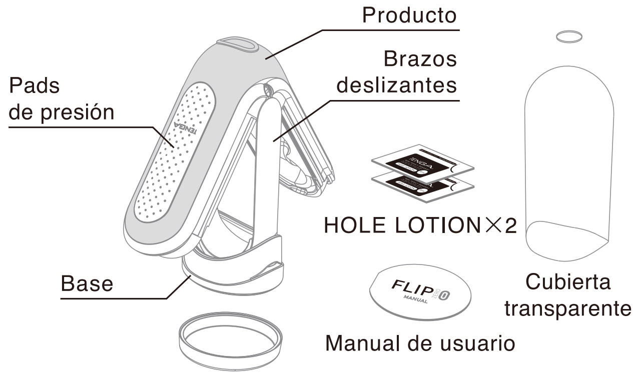

Apertura (1)

Retirez le bras coulissant

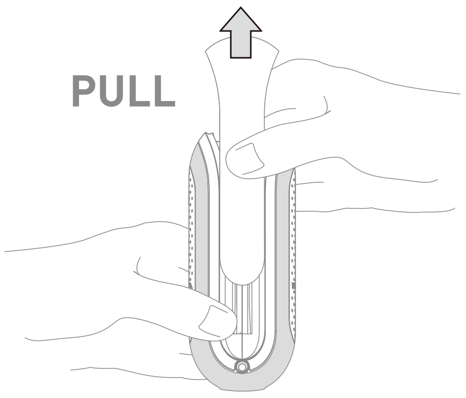

Apertura (2)

Aprieta las guías hasta que se suelte el bloqueo.

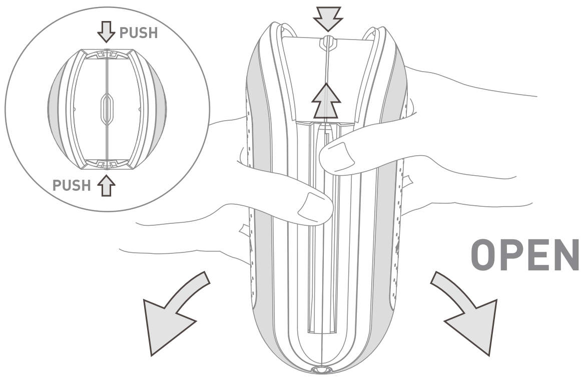

Antes del uso

Lubrica el interior de FLIP ZERO
Cierra el producto, bloquéalo con los brazos deslizantes y lubrica el punto de inserción.

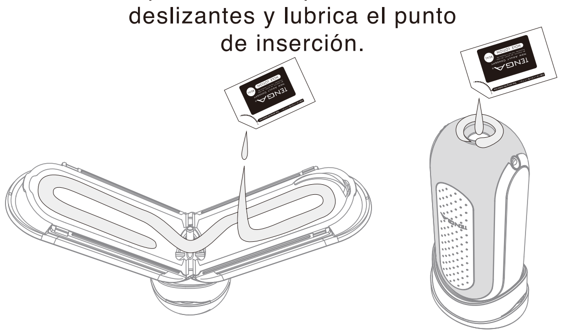

※Para usos futuros, recomendamos la marca HOLE LOTION de TENGA (se vende por separado).

Uso de los pads de presión

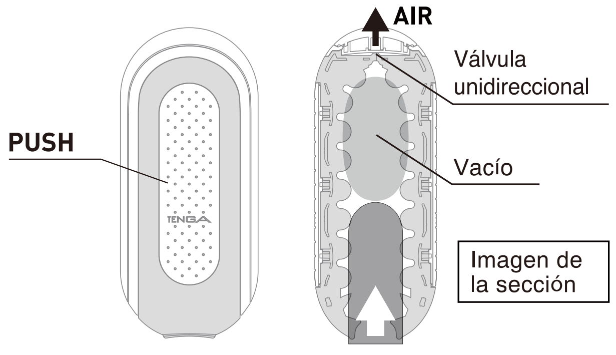

Tras la inserción, oprime cualquier punto de los pads de presión para centrar la estimulación en ese punto y crear un vacío interno.

Estructura interna

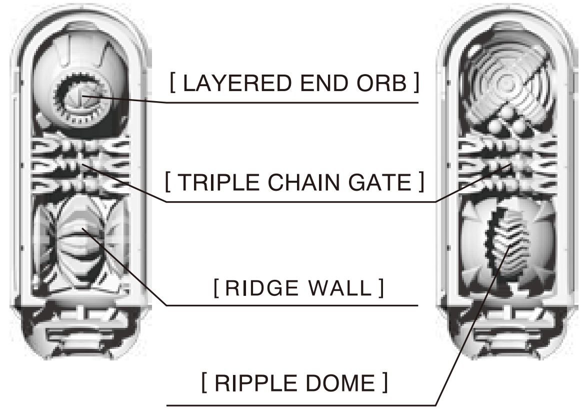

Secado

Retirez les bras coulissants et ouvrir l'accessoire.
Nettoyez soigneusement à l'eau chaude.

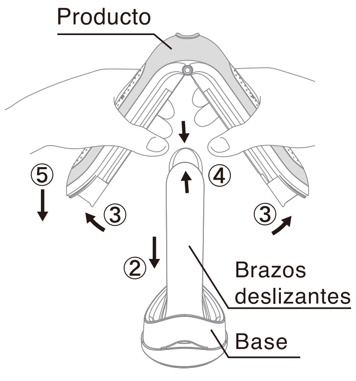

①Seca el producto con una toalla.
②Coloca los brazos deslizantes en la base.
③Sujeta FLIP ZERO con las dos manos.
④Aprieta los brazos deslizantes con el pulgar y el dedo corazón.
⑤Coloca FLIP ZERO en los brazos deslizantes

Almacenamiento

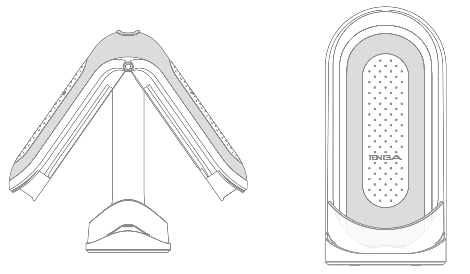

①Asegúrate de que el producto está completamente seco.
②Pon el producto cerrado en la base y coloca la cubierta superior.
③Guárdalo en un lugar fresco, seco y protegido de la luz del sol.

Advertencia de uso

Artículo para el placer masculino.
Guárdelo en un lugar fresco, seco y oscuro.
Manténgalo alejado de los niños.
Este artículo es una novedad.
TENGA Co., Ltd. no se hace responsable si este artículo es utilizado de forma distinta a su uso recomendado.
No lo comparta.
No lo utilice si su piel está dañada.
Para protégerlse de una posible irritación, pruebe una pequeña cantidad de lubricante en la parte interior de su brazo.
Si se produce irritación, lave con agua inmediatamente y consulte a su médico.

TENGA FLIP 0 (ZERO)

TFZ-001

Materials: Elastomer / PC / GPPS / ABS / Silicone
180×80×70mm / 480g

www.tenga-global.com/es

ANEXO 3

Programa de ejercicios SCT
FICHAS PACIENTES

En esta primera semana comenzaremos con lo que se denomina **Programa de Stop and Go Flip Hole.**

OBJETIVOS

1. Tomar conciencia del papel del suelo pelvico en la respuesta orgásmica.
2. Identificar el patrón de tensión muscular en la zona genital necesario para eyacular.
3. Identificar el carácter voluntario de ese patrón de tensión muscular que es necesario para eyacular .

CLAVES

Realizar la actividad solo una vez visto el video correspondiente a la actividad Descubriendo el Suelo Pelvico.

La actividad se realiza individualmente.

El hombre debe entender antes de realizar la actividad el contenido teórico de la misma.

Se realizará un mínimo de 4 veces o hasta que el hombre sea capaz de alcanzar los objetivos de esta primera semana.

No se trata de masturbarse sin más ,sino de una actividad de toma de c onciencia del papel del suelo pelvico.

ACTIVIDAD

Busque un momento de tranquilidad en el que no se le pueda interrumpir. Desconecte el teléfono, apaguen el televisor y la radio, no deje que los niños u otras personas que convivan con usted le distraigan.

Empezará a acariciar el pene, lenta y progresivamente, hasta que se provoque una erección, a partir de ahí comenzará a masturbarse utilizando el dispositivo Flip Zero al que previamente habrá aplicado unas gotas del lubricante adjunto en el kit, prestando atención a los cambios que se vayan produciendo alrededor de la zona genital y en la musculatura cercana.

Es importante que intente identificar como a medida que aumenta la excitación ,aumenta la tensión muscular en la zona genital y músculos cercanos a la misma.. Debe estar atento a identificar estos puntos de tensión o rigidez muscular en piernas, abdomen, glúteos y sobre todo suelo pélvico (debajo de los testiculos y en el esfinter anal).

Continúe masturbándose hasta eyacular y note como para poder llegar a provocar el reflejo eyaculatorio, es necesario no solo estimular el pene, sino también generar este patrón de tensión muscular en la zona, en especial en el suelo pélvico.

La tensión muscular en el suelo pélvico seria por tanto condición necesaria para eyacular y ademas de caracter voluntario, siendo este grupo muscular la llave que permite retardar o acelerar el reflejo eyaculatorio.
Si este grupo muscular ,conocido como suelo pelvico y en especial el esfinter anal y el de la uretra, no estan en tensión el reflejo de eyaculación no podrá darse.

Durante las próximas tres semana continuaremos con lo que se denomina **Programa de Star-Stop.**

OBJETIVOS

1. Conocer los cambios fisiológicos de un ciclo sexual masculino completo.
2. Identificar en el cuerpo los cambios en la musculatura de la zona genital de la fase preorgásmica.
3. Identificar el carácter voluntario de la tensión muscular que inicia la fase preorgásmica.
4. Empezar a desarrollar control sobre esta musculatura involucrada en la fase preorgásmica.

CLAVES

Realizar la actividad solo una vez haber identificado en la actividad 1 la tensión muscular en la zona genital previa a la eyaculación y entender por tanto el papel que juega el suelo pélvico en la respuesta orgásmica.

La actividad se realiza individualmente, intentando que su duración no sea inferior a 5 minutos.

El hombre debe entender antes de realizar la actividad el contenido teórico de la misma.

Se realizará tantas veces como sea necesario hasta que el hombre sea capaz de alcanzar los objetivos de de esta segunda actividad , se recomienda un mínimo de 4 veces por semana.

No se trata de masturbarse sin más, sino de una actividad de entrenamiento en autocontrol.

ACTIVIDAD

Busque un momento de tranquilidad en el que no se le pueda interrumpir. Desconecte el teléfono, apague el televisor y la radio, no deje que los niños u otras personas que convivan con usted invadan el dormitorio.

Empezará a acariciar el pene, lenta y progresivamente, hasta que se provoque una erección, a partir de ahí comenzará a masturbarse con el dispositivo Flip Zero lubricado prestando atención a los cambios que se vayan produciendo en la zona genital.

Es importante que intente identificar como a medida que aumenta la excitación ,aumenta la tensión muscular en la zona genital y músculos cercanos a la misma.. Debe estar atento a identificar estos puntos de tensión o rigidez muscular en piernas, abdomen, glúteos y sobre todo suelo pélvico.

Continúe masturbándose hasta que considere que le falta un minuto o dos para eyacular, en ese momento pare de estimularse e intente identificar como para poder llegar a provocar el reflejo eyaculatorio, ha generado un patrón de tensión muscular en la zona del suelo pélvico y alrededor de los genitales.

A continuación, antes de que pase un minuto desde que paro de estimularse, debe practicar el soltar la tensión de esa zona y dejarla relajada, con especial atención al esfínter anal y de la uretra, que quedaran sin tensión.

Cuando vuelva a estimular debería notar, si ha conseguido realmente soltar la tensión, que se aleja del umbral eyaculatorio y que ya no está tan cerca de eyacular.

Este proceso de parar y volver a estimular debe realizarlo al menos cuatro veces en cada actividad, terminando eyaculando dentro del dispositivo una vez realizadas las paradas esta vez si generando tensión en la musculatura de la zona genital.

En esta quinta semana continuaremos con lo que se denomina **Programa de Stop and Go sin cese de estimulación.**

OBJETIVOS

1. Conocer los cambios fisiológicos de un ciclo sexual masculino completo.
2. Identificar en el cuerpo los cambios fisiológicos de la fase preorgásmica.
3. Identificar el carácter voluntario de la fase preorgásmica.
4. Afianzar el control de la fase preorgásmica a través de la musculatura del suelo pelvico en condiciones similares al coito.

CLAVES

Realizar la actividad solo una vez entendido el papel que juega el suelo pélvico en la respuesta orgásmica,asi como habiendo alcanzado los objetivos de las semanas precedentes .

La actividad se realiza individualmente.

El hombre debe entender antes de realizar la actividad el contenido teórico de la misma y visualizado el video.

Se realizará tantas veces como sea necesario hasta que el hombre sea capaz de alcanzar los objetivos de esta tercera actividad, se recomienda un máximo de 5 actividades por semana.

No se trata de masturbarse sin más, sino de una actividad guiada de entrenamiento en autocontrol.

ACTIVIDAD

Busque un momento de tranquilidad en el que no se le pueda interrumpir. Desconecte el teléfono, apague el televisor y la radio, no deje que los niños u otras personas que convivan con usted invadan el dormitorio.

Empezará a acariciar el pene, lenta y progresivamente, hasta que se provoque una erección, a partir de ahí comenzará a masturbarse con el dispositivo Flip Zero suficientemente lubricado , prestando atención a los cambios que se vayan produciendo en la zona genital y alrededor.

Es importante que intente identificar como a medida que aumenta la excitación, aumenta la tensión muscular en la zona genital y músculos cercanos a la misma. Debe estar atento a identificar estos puntos de tensión o rigidez muscular en piernas, abdomen, glúteos y sobre todo suelo pélvico.

Continúe masturbándose hasta que considere que le falta un minuto o dos para eyacular si sigue asi, en ese momento y sin dejar de estimular el pene con el masturbador y sin cambiar el ritmo, céntrese en su patrón de tensión muscular en la zona del suelo pélvico y alrededor de los genitales.

A continuación, y repetimos sin dejar de estimular, practique el soltar la tensión de esa zona y dejarla relajada, con especial atención al esfínter anal y el de la uretra, que quedaran sin tensión, para ello puede probar hacer el gesto de inicio de la micción que relaja estos esfínteres de forma voluntaria.

Si ha conseguido soltar la tensión, notará que se aleja del reflejo eyaculatorio y que ya no está a uno o dos minutos de eyacular. Pero recuerde sin dejar de estimular todo el tiempo con el dispositivo.

Este proceso de retardar el reflejo eyaculatorio debe realizarlo al menos cuatro veces en cada actividad de masturbación, terminando eyaculando esta vez si, forzando la tensión muscular , una vez realizada la actividad.

En esta septima semana continuaremos con lo que se denomina **Programa de Star-Stop Zero con pequeñas paradas y con movimientos de cadera similares a la penetración.**

OBJETIVOS

1. Conocer los cambios fisiológicos de un ciclo sexual masculino completo.
2. Identificar en el cuerpo los movimientos de cadera sin necesidad de tensar suelo pelvico.
3. Identificar el carácter voluntario de la tensión muscular previa de la fase preorgásmica.
4. Afianzar el control de la fase preorgásmica a través de la musculatura del suelo pelvico en condiciones similares al coito y con movimientos de cadera .

CLAVES

Realizar la actividad solo una vez entendido el papel que juega el suelo pélvico en la respuesta orgásmica,asi como habiendo alcanzado suficientmentes los objetivos de las semanas precedentes .

La actividad se realiza individualmente y sin mover los brazos para masturbarse.

El hombre debe aprender a mover la cadera sin tensar la musculatura del suelo pelvico .

Se realizará tantas veces como sea necesario hasta que el hombre sea capaz de alcanzar los objetivos de esta cuarta semana, se recomienda un máximo de 5 actividades por semana.

No se trata de masturbarse sin más, sino de una actividad guiada de entrenamiento en autocontrol muscular.

ACTIVIDAD

Busque un momento de tranquilidad en el que no se le pueda interrumpir. Utilice material audiovisual que le provoque cierta distracción y que sea de contenido sexual con el fin de alcanzar un mayor nivel de excitación ,asi como una mayor dificultad para percibir sensaciones internas.

Empezará a acariciar el pene, lenta y progresivamente, hasta que se provoque una erección, a partir de ahí, comenzará a masturbarse con el dispositivo Flip Zero suficientemente lubricado , prestando atención a su propio cuerpo, esto debe hacerse de pie o de rodillas y empujando con movimientos de cadera contra el dispositivo, sin mover la mano, como en la penetración.

Continúe masturbándose hasta que note la sensación de que le falta un minuto o dos para eyacular , en ese momento sin sacar el pene del masturbador , céntrese en su patrón de tensión muscular en la zona del suelo pélvico y alrededor del esfinter anal ,con especial atención a los gluteos.

A continuación, practique el soltar la tensión de esa zona y dejarla relajada, con especial atención al esfínter anal , gluteos y musculatura suelo pelvico, que quedaran sin tensión, para ello puede probar hacer el gesto de inicio de la micción que relaja estos esfínteres de forma voluntaria.

Si ha conseguido soltar la tensión, notará que se aleja del reflejo eyaculatorio y que ya no está a uno o dos minutos de eyacular. Pero recuerde sin sacar el pene del dispositivo lubricado. La clave del control pasa por mover la cadera como si tuvieramos un hula-hoop, pudiendo desarrollar velocidad sin tensión.

Este proceso de retardar el reflejo eyaculatorio debe realizarlo tantas veces como sea necesario en cada actividad de masturbación, ya que la duración total del ejercicio no debe ser inferior a 15 minutos terminando eyaculando esta vez si forzando la tensión muscular en gluteos y esfinter anal una vez realizada la actividad.
